# Characterisation and *In Planta* Activity of Bacteriophages That Infect the Key Bacterial Species Associated With Acute Oak Decline

**DOI:** 10.1111/1751-7915.70394

**Published:** 2026-06-02

**Authors:** Emily R. Grace, Vanja Milenkovic, Katherine G. Hinton, Sabrine Dhaouadi, Diana Vinchira‐Villarraga, Michael A. Brockhurst, Mojgan Rabiey, Robert W. Jackson

**Affiliations:** ^1^ The Birmingham Institute of Forest Research and School of Biosciences University of Birmingham Birmingham UK; ^2^ Division of Evolution, Infection and Genomic Sciences, School of Biological Sciences, Faculty of Biology, Medicine and Health The University of Manchester Manchester UK; ^3^ School of Life Sciences University of Warwick Coventry UK

**Keywords:** bacteriophage, plant pathogens, *Quercus* spp.

## Abstract

Acute oak decline (AOD) is a complex disease of oak trees (*Quercus* spp.) which results in bleeding stem cankers and potential tree mortality. A polymicrobial complex is implicated in causing AOD, of which the most commonly isolated species are *Brenneria goodwinii* and *Gibbsiella quercinecans* . Bacteriophages (phages), natural predators of bacteria, can act as biocontrol agents that reduce bacterial infections in trees. In this study, the first phages that lyse *B. goodwinii* and *G. quercinecans* , BREN6 and GIB1 phages respectively, were isolated from AOD‐impacted trees. Genomic analyses and transmission electron microscopy showed that BREN6 is a novel phage type with siphovirus morphology, whereas GIB1 possesses characteristics typical of N4‐like phages. While in vitro assays showed that each phage could effectively reduce their hosts' population growth for at least 24 h, application of the phages to either single or dual species communities of *B. goodwinii* and *G. quercinecans* in the stems of oak saplings showed that the phages had a minimal impact on their host populations, and in some trees actually resulted in higher bacterial populations. Our data show that the application of these phages does not alter the *in planta* population dynamics between *B. goodwinii* and *G. quercinecans* , and emphasises the importance of assessing phage effectiveness within natural settings.

## Graphical abstract

Phages that infect the oak pathogens *Brenneria goodwinii* and *Gibbsiella quercinecans* were isolated. The phages were able to effectively reduce bacterial populations of their respective hosts in vitro. However, when applied *in planta*, the phages did not reduce host populations and instead phages and bacteria co‐existed over time.

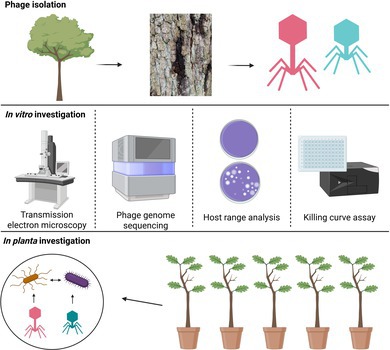

## 1 Introduction

Oak trees (*Quercus* spp.) across Europe are currently experiencing a significant downturn in health. One of the main causes is acute oak decline (AOD), a condition characterised by abiotic stress that reduces tree vigour and leads to the onset of bleeding cankers in the tree stems. Together, these symptoms can reduce canopy size and potentially lead to tree mortality within 3–5 years of initial symptoms (Denman et al. 2014). As a decline disease, AOD is instigated by multiple co‐occurring abiotic and biotic factors. Associated abiotic factors include soil water saturation and poor nutrient availability, which cause a tree to enter a declining state (Brown et al. 2018; Shaw et al. 2025). Notably, oak trees showing disease symptoms display differences in the production of secondary metabolites indicating a possible driver for changes in microbial dynamics (Vinchira‐Villarraga et al. 2025). The most prominent biotic factor impacting tree health is the infection of the stems by a polymicrobial consortium of Gram‐negative bacteria that cause bleeding cankers (Brady et al. 2017; Denman et al. 2018). The bacterial species most frequently isolated within the polymicrobial consortia of AOD‐impacted trees are *Brenneria goodwinii* (*Bg*), *Gibbsiella quercinecans* (*Gq*), *Rahnella victoriana* (*Rv*) and *Lonsdalaea britannica* (Brady et al. 2010, 2014, 2012; Denman et al. 2012).

*Bg* and *Gq* are most often implicated in causing bleeding cankers in trees suffering from AOD. Both species have been isolated from canker tissues of various *Quercus* species across Europe and Iran, including *Q. robur* (pedunculate oak), *Q. petraea* (sessile oak), *Q. ilex* (holm oak) and *Q. cerris* (Turkey oak) (Carluccio et al. 2024; Denman et al. 2018; Ruffner et al. 2020). *Gq* has also been isolated from declining *Juglans regia* (common walnut) and *Elaeagnus angustifolia* (black olive) in Iran, as well as *Tilia cordata* (small‐leaved lime) in Poland (Allahverdipour et al. 2021; Basavand et al. 2021; Tkaczyk et al. 2024). Genomic characterisation and inoculation studies of these bacteria have shown that both bacterial species are likely opportunistic pathogens stimulated by the decline of tree health, or by the presence of the two‐spotted oak buprestid beetle *Agrilus biguttatus* (Broberg et al. 2018; Cambon et al. 2024; Denman et al. 2018). Transcriptomic analysis of bacteria isolated from diseased and asymptomatic trees showed the upregulation of virulence factors, including some genes associated with biofilm formation (Broberg et al. 2018). This may indicate biofilm structures are produced at some time during disease development, creating a more complex and protected microbial environment.

There is mounting evidence that *Bg* and *Gq*, or possibly combinations of either pathogen with other members of the AOD‐associated polymicrobial consortia, interact together to form and maintain bleeding cankers. Denman et al. (2018) demonstrated that inoculation of oak logs with both *Bg* and *Gq* resulted in the formation of significantly larger cankers compared to those that formed when either bacterium was inoculated alone. *Bg* has also been shown to differentially express genes dependent on the presence of *Gq* (Doonan et al. 2020). For example, *Bg* upregulated genes related to sugar and iron transport, and plant cell wall‐degrading enzymes, only when grown alongside *Gq*, suggesting that *Gq* aids the necrosis of oak tissue by *Bg*. Brady et al. (2022) showed that passaging of *Bg* with either *Gq* or *Rv* resulted in an increase in the in vitro growth of *Bg*, demonstrating that interaction with other bacteria enhances the fitness of *Bg*.

Accordingly, the AOD microbiome is complex, and significant changes in bacterial population dynamics and diversity are likely to occur during the shift from a healthy to a diseased oak microbiome (Denman et al. 2016). One key and often overlooked influence on plant microbiomes, and subsequently on plant health, is that of bacteriophages. Bacteriophages (phages) are viruses that infect and kill bacteria. They can play a significant role in maintaining bacterial diversity in natural microbiomes. For example, phage populations often infect the most common bacterial species within a microbiome, preventing domination by a single or a few species (‘kill the winner’ hypothesis) (Brockhurst and Koskella 2013; Chevallereau et al. 2022). Moreover, the presence of phages that target bacterial pathogens within a microbiome can also be key to plant health. Yang et al. (2023) demonstrated that increased abundance of phages capable of infecting the pathogen *Ralstonia solanacearum* in the tomato microbiome resulted in fewer diseased plants. In turn, the lack of these phages, along with an increase of phages that lyse the disease‐suppressing bacterium *Stenotrophomonas maltophilia* , resulted in more diseased plants.

The lytic abilities of phages also render them as potential biocontrol agents against bacterial plant diseases. A single phage can often only infect a single bacterial species, and this specificity makes them desirable in comparison to broad‐spectrum antimicrobials, such as antibiotics and metal compounds. Over the past decade, there have been several successes in using phages to treat bacterial tree diseases. Phages can be used commercially to control several bacterial tree diseases, including Erwiphage PLUS and Agriphage‐Fire Blight against *Erwinia amylovora* , and XylPhi‐PD against *Xylella fastidiosa* (Grace et al. 2021). Extensive research has also been conducted to isolate and characterise phages of other important bacterial tree diseases, such as *Pseudomonas syringae* pathovars *syringae* and *actinidae*, which cause canker of *Prunus* species and kiwi, respectively (Frampton et al. 2014; Pinheiro et al. 2020; Rabiey et al. 2020, 2024).

To our knowledge, no lytic phages have yet been isolated against *Bg* or *Gq*, despite their importance as oak tree pathogens. The aim of this study was therefore to isolate phages that infect these two species and investigate their key biological characteristics, including their ability to prevent the growth of their hosts in vitro and *in planta*. This will help to reveal the phages' suitability as potential biocontrol agents and further understand the role of phages in the AOD microbiome.

## 2 Methods

### 2.1 Bacterial Strains and Culture Conditions

*Bg* strains FRB 141^T^ and FRB 171, and *Gq* strains FRB 97^T^ and FRB 124, were used to isolate phages from the environment. Bacterial strains were kindly provided by Dr. Carrie Brady from the University of West of England, UK. Bacterial cultures were grown in nutrient broth (NB; Oxoid, UK) or nutrient agar (NA; Oxoid). Soft agar overlay (1:1 ratio of molten NA and sterile water) was used for plaque and spot assays. Both bacterial species were maintained as glycerol stocks at −80°C. Liquid cultures of each species were produced by inoculating strains into 5 mL of NB and incubating overnight at 27°C with shaking at 180 rpm.

### 2.2 Sample Collection

Sampling for phages took place at two oak woodlands in the Midland region of England: Norbury Park Estate, Staffordshire (52°47′40.6″ N, 2°17′53.9″ W); and Wyre Forest, Worcestershire (52°22′31.7″ N, 2°21′09.8″ W). Both forests contain large areas of oak stands, at least 50 years old, which have been historically managed for commercial purposes. All samples were taken from mature *Quercus robur* specimens. Trees were classed as impacted by AOD if they displayed typical AOD symptoms, namely longitudinal weeping stem cankers, D‐shaped exit holes and canopy dieback (Denman et al. 2014). At least three healthy and three infected trees were sampled at each site.

Soil samples were taken from three points using a soil borer with a 10 cm diameter in a 1 m radius around the base of each tree, at a depth of approximately 5 cm. Leaf samples were taken at three different points around the lower canopy of each tree using an extendable tree pruner. 2 cm × 2 cm sections of bark and canker tissues were taken from each healthy and diseased tree using a chisel and mallet. All tools were sterilised with 70% ethanol between each use. Samples were stored at 4°C following collection.

### 2.3 Sample Processing for Bacteria Isolation

To isolate *Bg* and *Gq* from each sample site, four chips of bark from each diseased and healthy sample were placed onto MacConkey agar plates (Oxoid; MacConkey 1900). Where trees had actively bleeding cankers, cotton swabs were taken of the exudate and applied to plates. MacConkey agar selects for Gram‐negative enteric bacteria and was chosen as both *Bg* and *Gq* produce recognisable differential colony morphology types on it. Plates were incubated for 2 days at 27°C, after which colonies were picked and restreaked. Frozen glycerol stocks of each colony were prepared using 800 μL of an overnight culture of each colony in NB and 800 μL of 40% sterilised glycerol. A colony PCR targeting the *gyrB* gene of *Bg* and *Gq* was performed on each colony for identification. For *Bg* identification, a forward primer of 5′‐TCGACGAGCGCGTAAAAGAT‐3′ and a reverse primer of 5′‐CACTGCAAAGCCACTTCCAC‐3′ were used, producing a 160 bp product. PCR conditions were as follows: denaturation at 95°C for 2 min; 30 cycles of denaturation at 95°C for 30 s, annealing at 55.9°C for 1 min and extension at 72°C for 1 min; and a final extension at 72°C for 10 min. For identifying *Gq*, primers and conditions were used as described in Pettifor et al. (2020).

### 2.4 Sample Processing for Phage Isolation

All sample types were processed by homogenising approximately 0.2 g of each sample in 1.5 mL of phosphate‐buffered saline (PBS; Oxoid) with two metal beads (Fisher Scientific, USA) at 4 m s^−1^ for 20 s. Samples were centrifuged (30 s, 14,000 rpm), and 100 μL supernatant was used in a plaque assay by mixing with 5 mL soft agar containing 100 μL of each bacterial strain (OD600 = 0.5). The mixture was overlaid onto agar plates, incubated overnight at 27°C and examined for plaques (zones of lysis) the following day.

Where plaques formed, agar plugs were collected using a 200 μL pipette tip and resuspended in 1 mL PBS. The suspension was replated, and a single plaque was picked. This process was repeated four times to ensure a single phage isolate. Phage stocks for future work were produced by performing plaque assays using phage samples with a titre of 10^6^–10^8^ plaque forming units per millilitre (PFU mL^−1^), and washing the plate surface with 5 mL of PBS. Phages were then collected from the plates using a 5 mL syringe and passed through a sterile 0.22 μm filter (Millipore, Ireland). The titre of each phage stock was determined by spotting serial dilutions of each phage stock onto soft agar containing the required host. Following incubation at 27°C overnight, plaques were counted at each dilution to calculate plaque forming units per millilitre (PFU mL^−1^). Phage stocks were stored at 4°C.

### 2.5 Bacterial Genome Analysis

Bacterial strains that had not been previously sequenced were sent directly to MicrobesNG, Birmingham, UK (https://www.microbesng.com/), for hybrid sequencing. Strains were prepared using MicrobesNG's standard strain submission protocol. Hybrid sequencing was performed using Illumina short‐read sequencing on a NovaSeq 6000 using 2 × 250 bp kits, and Oxford Nanopore long‐read sequencing on a GridION with R10.4.1 flow cells. Reads were checked for quality using FastQC (Andrews 2010). Bacterial genomes were assembled using Unicycler (Wick et al. 2017), annotated using Prokka (Seemann 2014) and visualised using Geneious Prime v2025.1.2.

### 2.6 Phage Genome Analysis

DNA was extracted from phages infecting *Gq* using the Norgen Biotek Phage DNA Isolation Kit (Ontario, Canada). Extraction was performed following the manufacturer's instructions using high‐titre phage samples (10^8^–10^10^ PFU mL^−1^). As phages infecting *Bg* could not be extracted using the kit, a phenol‐chloroform extraction method was employed instead (https://www.protocols.io/view/phage‐dna‐extraction‐with‐phenol‐chloroform‐and‐di‐8epv5jrxnl1b/v1?step=13). Phage suspensions at 10^8^–10^9^ PFU mL^−1^ were combined with 1 μL proteinase K and 0.05% SDS and incubated at 55°C for 1 h with shaking at 300 rpm. The DNA was then extracted using phenol‐chloroform‐isoamyl alcohol (49.5:49.5:1; Sigma‐Aldrich, UK) and chloroform. The DNA was precipitated overnight at 4°C in isopropanol and 3 M sodium acetate, after which it was centrifuged for 30 min at 14,000 rpm. The DNA pellet was washed twice with 70% ethanol solution (v/v) and resuspended in 30 μL of nuclease‐free water. DNA quantity was measured using a Qubit 4 Fluorometer (ThermoFisher Scientific, UK) with a dsDNA broad range assay kit. DNA quality was measured using a NanoDrop (ThermoFisher Scientific).

Phage genomes were sequenced by MicrobesNG (Birmingham, UK) using Illumina sequencing with 30× coverage. Paired short reads were trimmed using Trimmomatic v0.30, and quality was assessed using FastQC v0.11.9 (Bolger et al. 2014). Genomes were assembled *de novo* with Shovill (https://github.com/tseemann/shovill), using SPAdes v3.14.1 as the assembler (Prjibelski et al. 2020). Complete phage contigs were identified via Bandage (Shen and Millard 2021; Wick et al. 2015). Contigs were then annotated using Pharokka v1.2.0 (Bouras et al. 2023), with PHANOTATE used to predict coding sequences (McNair et al. 2019) and reoriented using the large terminase (TerL) subunit. Phage taxonomy was identified using BLASTn and tax_myPHAGE (https://github.com/amillard/tax_myPHAGE). To identify any potential antimicrobial resistance or virulence genes, genomes were placed through the web server PhageLeads (Yukgehnaish et al. 2022).

### 2.7 Transmission Electron Microscopy

To visualise phage morphology, transmission electron microscopy (TEM) was performed. High‐titre phage stocks with minimal bacterial debris were prepared using the phage enrichment and PEG precipitation methods outlined in Rabiey et al. (2020). TEM took place at the Midlands Regional Cryo‐EM Facility at the University of Warwick. A 10 μL volume of each phage stock was applied to a carbon/formvar TEM grid (EM resolutions, UK) and stained with 2% uranyl acetate. Images were taken using a JEOL 2100Plus microscope equipped with a Gatan Ultrascan camera.

### 2.8 One‐Step Growth Curves

To determine the growth kinetics of the phages, one‐step growth curves were performed (Hyman and Abedon 2009). *Bg* strain FRB 141^T^ and *Gq* strain FRB 124 were used to perform growth curves of phages BREN6 and GIB1, respectively. Cultures of each bacterium were grown at 27°C in NB with shaking at 180 rpm for 3–4 h. A 900 μL volume of each bacterial culture was added to 100 μL of phage (10^7^ PFU mL^−1^) and incubated at 27°C for 5 min. The mixtures were then spun down and resuspended in 1 mL of NB to remove detached phages. The suspension was added to 9 mL of prewarmed broth and incubated at 27°C with shaking. Samples were removed immediately (time 0), with subsequent sampling taking place every 10–15 min for up to 165 min. Three replicate experiments were performed for each phage. A preliminary growth curve assay was performed for each phage type to determine ideal sampling times. Burst size was determined by dividing the average PFU mL^−1^ value throughout the latent period by the average of the final three PFU mL^−1^ values of the experiment for each replicate (Zhao et al. 2019).

### 2.9 Host Range Determination

The host range of each phage was determined to assess their specificity. All species tested are outlined in Table S1. Each bacterial isolate was adjusted to an OD_600_ of 0.5 and applied to soft agar overlay. Five microlitre of each phage (10^6^ PFU mL^−1^) was then spotted in triplicate onto the agar surface. Plates were incubated overnight at 27°C and checked for plaques the following day. The experiment was performed twice independently.

### 2.10 Identification of Phage Defence Systems Within Different *Bg* and *Gq* Strains

To identify phage defence systems, bacterial genomes were analysed using the Prokaryotic Antiphage Defence LOCator (PADLOC) web server v2.0.0, with CRISPRDetect to determine CRISPR arrays (Biswas et al. 2016; Payne et al. 2022). Prophage sequences were identified using PHASTEST (Wishart et al. 2023). Overlaps of phage defence and prophage sequences in bacterial genomes were assessed using Geneious Prime v2025.1.2.

### 2.11 Temperature Sensitivity Assays

A temperature sensitivity assay was performed to determine the ideal temperature to store the phages at to preserve their titres. This was performed by placing 1 mL of phage stocks of a known titre (10^5^–10^7^ PFU mL^−1^) in 2 mL Eppendorf tubes at different temperatures (−20°C, 4°C, 20°C, 27°C and 37°C). Tubes were wrapped in parafilm to prevent evaporation and covered with metal foil to prevent degradation from UV irradiation. Each stock was titred in triplicate via a spot assay after 1 week (short‐term storage) and 1 year (long‐term storage). Three replicate populations were tested for each phage at each temperature, with three technical replicates recorded per population.

### 2.12 Killing Curve Assays at Different Multiplicities of Infection

Killing curve assays using different ratios of phages to bacterial cells, known as multiplicity of infection (MOI), were performed to determine the most effective phage titre required to reduce bacterial growth. To test the phage infecting *Bg* (BREN6), MOIs of 0.1, 0.01 and 0.001 were performed using *Bg* FRB 141^T^ at an OD_600_ of 0.5 (~10^9^ CFU mL^−1^) and phages at titres of 10^8^, 10^7^ and 10^6^ PFU mL^−1^, respectively. For the phage infecting *Gq* (GIB1), MOIs of 0.01, 0.001 and 0.0001 were performed using *Gq* FRB 124 at an OD_600_ of 0.5 (~10^9^ CFU mL^−1^) and phages at 10^7^, 10^6^ and 10^5^ PFU mL^−1^, respectively. One hundred microlitre volumes of each phage and their respective bacteria were applied to a 96‐well plate. Negative controls included each bacterium with PBS; each phage stock with NB; and PBS with NB. The plate was placed into a TECAN SPARK Multimode Microplate Reader, and absorbance of wells at 600 nm (A600) was measured every 20 min for 48 h at 27°C. Three replicate populations were tested for each MOI.

### 2.13 Inoculation of Oak Saplings

To investigate the interaction between *Bg*, *Gq* and their phages *in planta*, both bacterial species and their respective phages were inoculated into oak saplings. Two separate inoculation studies were conducted using either large (2 m) field‐grown saplings or small (30–50 cm) cell‐grown saplings. All saplings were *Q. robur* and approximately 2 years old. Saplings were kept within a temperature‐controlled glasshouse, with the temperature set at 22°C for 16 h during the day and at 16°C for 8 h during the night. Large and small saplings were transferred into 12 and 2 L pots, respectively, with a 1:4 perlite and compost mix, treated with Solufeed fertiliser (Chichester, UK), and watered twice weekly.

Bacterial inocula for each experiment were prepared by producing overnight cultures from single colonies of *Bg* FRB 141^T^ and *Gq* FRB 124 in 20 mL of NB. Cultures were adjusted to an OD_600_ of 0.5, washed twice and resuspended in PBS. High titre (~10^9^ CFU mL^−1^) stocks of BREN6 and GIB1 were prepared using the PEG precipitation method, and their titre was determined prior to application via spot assay. In treatments containing both bacteria or both phages, equal volumes of both bacteria or both phage stocks were mixed and vortexed for 30 s prior to inoculation.

To track the population dynamics of phages and bacteria over time in oak stem tissue, large oak saplings were inoculated with *Bg* and *Gq*, either with or without BREN6 and GIB1. Three days prior to inoculation, a 3 cm × 2 cm sample of bark was removed from each large oak sapling using a sterile surgical scalpel to determine the baseline level of Gram‐negative bacteria pre‐existing in the trees. On the day of inoculation, four 3 × 2 cm longitudinal wounds were made along the stem of each large oak sapling using a surgical scalpel. Twenty trees were inoculated in total, and all wounds on five trees each were inoculated with one of the following treatments: ‘*Bg* + *Gq* only’; ‘BREN6 + GIB1 only’; ‘*Bg* + *Gq* + BREN6 + GIB1’ and ‘control’ (sterile water). For the first treatment, 50 μL of either bacterial inoculum (‘*Bg* + *Gq* only’; ‘*Bg* + *Gq* + BREN6 + GIB1’) or sterile water (‘BREN6 + GIB1 only’; ‘control’) was applied and allowed to dry for 30 min. Then, 50 μL of either the phage treatment (‘BREN6 + GIB1 only’; ‘*Bg* + *Gq* + BREN6 + GIB1’) or sterile water (‘*Bg* + *Gq* only’; ‘control’) was applied and again left to dry. Wounds were then wrapped with parafilm. For bacteria and phage quantification, a 3 cm × 2 cm sample was removed from a randomly selected wound on each tree immediately following inoculation (0 dpi), and at 5, 10 and 30 dpi (days post‐inoculation).

To test if the presence of one bacterium‐phage pair (i.e., *Bg* and BREN6) had an impact on the interaction between another bacterium‐phage pair (i.e., *Gq* and GIB1), 10 small saplings were inoculated with one of 12 different combinations of phages and bacteria. A total of 120 saplings were investigated. Single phage‐bacteria combinations were: ‘*Bg* only’; ‘*Gq* only’; ‘*Bg* + *Gq* only’; ‘*Bg* + BREN6’; ‘*Gq* + GIB1’. Multispecies combinations were: ‘*Bg* + *Gq* only’; ‘*Bg* + *Gq* + BREN6’; ‘*Bg* + *Gq* + GIB1’; ‘*Bg* + *Gq* + BREN6 + GIB1’. The ‘control’ treatment involved applying only PBS twice to the stem wound. Single 1 cm × 2 cm wounds were made on each sapling using a sterile scalpel. Then, 20 μL of the bacterial or control (PBS) treatment was applied to the wounds and allowed to dry for 30 min. A 20 μL volume of the phage or control treatment was then applied, allowed to dry for 30 min and wrapped in parafilm. The entire stem section spanning the wound site was taken from half of all trees immediately after application (0 dpi) for bacteria and phage quantification, and that of the remaining half was removed at 14 dpi.

All stem tissue samples were cut into three pieces using sterile scissors, placed in a homogenisation tube containing 1 mL PBS with a metal bead, and homogenised at 4 ms^−1^ for 30 s. The bacterial density of each sample was quantified by serially diluting the homogenate in PBS and plating the dilutions on MacConkey agar plates. Bacterial colonies on the plates were counted after 24 and 48 h of incubation at 27°C. To quantify BREN6 and GIB1 densities, 1 mL of each homogenised sample was passed through a 0.22 μm PES filter and serially diluted in PBS. Three microlitre of each dilution was spotted in triplicate onto NA plates overlaid with soft agar containing *Bg* FRB 141^T^ or *Gq* FRB 124, respectively. After 24 h, plaques were counted at each dilution. The remaining unfiltered homogenised samples were combined with an equal volume of 40% glycerol and stored at −80°C for future use.

To determine if *Bg* or *Gq* could develop phage‐resistance *in planta*, three individual colonies of *Bg* and *Gq* were isolated from each sample treated with ‘*Bg* + *Gq*’ and ‘*Bg* + *Gq* + BREN6 + GIB1’ during the large sapling experiment. Colonies were picked and restreaked three times to isolate single colonies and separate associated phages. The identity of each colony was confirmed by colony PCR using *Bg* and *Gq* specific primers and conditions described earlier. To test their phage susceptibility, *Bg* isolates were tested against BREN6, and *Gq* isolates against GIB1, using a 24‐h killing curve assay with phages at a concentration of 10^6^ PFU mL^−1^ following the same method described previously. Isolates were regarded as susceptible if they were unable to grow within 24 h after phage exposure.

### 2.14 Statistical Analyses

All bacterial CFU mL^−1^ and phage PFU^−1^ mL measurements were transformed by log_10_ + 1. To determine normality and homoscedasticity of all data, Shapiro–Wilk and Levene's tests were first performed in GraphPad Prism v10, with data classed as parametric and having equal variance if *p* > 0.05.

As data from the killing curve, temperature and first *in planta* assay were non‐parametric and contained repeat measures data, the impact of independent variables was assessed using general linear mixed models (GLMM) in RStudio v4.4, with replicate or population number classed as a random effect, using the R package ‘glmmTMB’ (https://glmmtmb.github.io/glmmTMB/). Bacterial CFU counts were analysed using a GLMM with a Gamma distribution and log link. Phage PFU counts were analysed using a GLMM with a Poisson distribution due to the presence of zero values (i.e., absence of plaques). Type III Wald Chi‐square tests and Tukey post hoc testing (95% confidence interval) was performed using packages ‘car’ (Fox and Weisberg 2019) and ‘emmeans’ (https://github.com/mjskay/ARTool), respectively. A non‐parametric aligned ranks transformation analysis of variance (ART ANOVA) and Tukey post hoc tests were performed in RStudio v4.4 using the packages ‘ARTool’ and ‘emmeans’, on the second *in planta* experiment to assess the impact of time and treatment on bacterial CFU and phage PFU.

## 3 Results

### 3.1 *Bg* and *Gq* Isolates Were Obtained From Both Healthy Bark and Canker Samples of Some Trees in Both Sampling Sites, Whereas Phages Were Only Isolated From Canker Samples

Three healthy (nor_H1, nor_H2, nor_H3) and three diseased trees (nor_D1, nor_D2, nor_D3) in Norbury Park, and five healthy (wyre_H1 to wyre_H5) and five diseased trees (wyre_D1 to wyre_D5) in Wyre Forest were chosen for sampling (Figure S1). Bark and canker samples were tested for the presence of *Bg* and *Gq* using species‐specific PCR. Two *Bg* strains were isolated from the canker tissue of diseased tree wyre_D4 (*Bg* strains J4.2 and J4.3). One *Bg* strain was isolated from the canker tissue of tree wyre_D3 (*Bg* W14) and the healthy bark tissue of wyre_H4 (*Bg* W19), respectively. One *Gq* strain was isolated from the diseased tissue of trees nor_D3 (*Gq* Norbury) and wyre_D1 (*Gq* W3), and from healthy tissue of wyre_H5 (*Gq* W15), respectively. Isolation of the bacteria demonstrated that the target bacteria were present at the sampling sites.

To isolate phages infecting *Bg* and *Gq*, bark, leaf and soil samples were collected from all trees and tested for phage presence using the bacterial strains *Bg* FRB 141^T^, *Bg* FRB 171, *Gq* FRB 97^t^ and *Gq* FRB 124. Multiple phages, which could lyse *Bg* strain FRB 141^T^ and were named BREN phages, were retrieved from canker tissue of two diseased trees (wyre_D2 and wyre_D4) in Wyre Forest, Worcestershire, in September 2022. The two trees were almost 650 m apart within the forest. Plaques of all the phages were circular and clear with a diameter of 1 mm (Figure S2a). Nine phages, named BREN1 to BREN9, were randomly selected for further study.

Multiple phages were isolated against *Gq* FRB 124 from diseased canker tissue of trees nor_D1 and nor_2 in Norbury Park, Staffordshire, in January 2022. These two trees were adjacent to each other (~2 m apart). All phages produced clear circular plaques with a diameter of ~1.5 mm on soft agar containing *Gq* FRB 124 (Figure S2b). From these initial isolation plates, five phages, named GIB1–GIB5, were randomly picked for further study. No phages against either bacteria were isolated from any healthy bark, soil or leaf sample at either location.

### 3.2 Phage Characterisation

#### 3.2.1 Genomic Analysis

**FIGURE 1:**
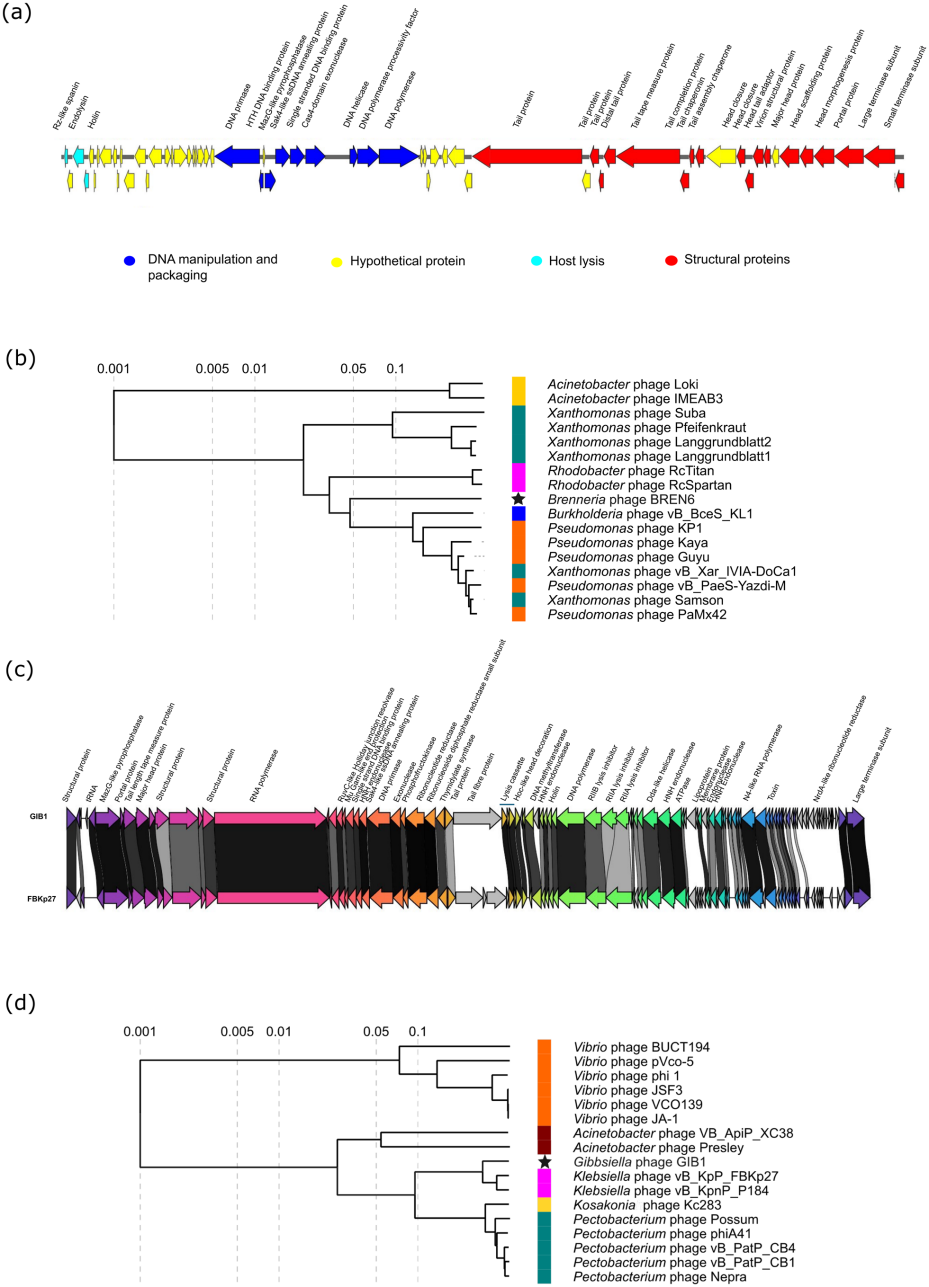
Genomic analysis of *Bg* phage BREN6 and *Gq* phage GIB1. (a) Visualisation of the genome of the phage BREN6 was made using SnapGene v7.2 (snapgene.com). (b) Proteomic tree of BREN6 and related phages extracted from the Virus‐Host database were made using ViPTree v4.0. Values represent genomic similarity scores (SG) of the BREN6 genome to the listed genomes, where 1 indicates identical and 0 indicates no similarity. Square colours correspond to host species. (c) Visualisation of the genome of the phage GIB1 and the *K. pneumoniae* infecting phage FBKp27 was made using Clinker v0.0.28. Amino acid percentage identity is represented by greyscale linkages between genomes. Arrows are placed in the direction of the transcription. Predicted protein function was assigned by Pharokka (Bouras et al. 2023). (d) Proteomic tree of GIB1 and related phages extracted from the Virus‐Host database, made using ViPTree v4.0.

DNA extraction and genome sequencing of all isolated phages were performed for genomic characterisation, and to identify differences between phages of the same host. The ICTV guidelines state that viral sequences with > 97% nucleotide identity can be considered the same phage species (Turner et al. 2021). Accordingly, all nine BREN phages isolated had 100% nucleotide identity and can therefore be classified as the same phage type. One phage, BREN6, was chosen as a representative of these phages for further experiments. The genome sequence of BREN6 is available under GenBank accession number PQ031257. BREN6's genome is 42,860 base pairs (bp) long and has a GC content of 40.5%. A total of 62 genes were predicted by the phage annotation programme Pharokka, with 18 genes related to DNA binding and packaging, three genes related to host lysis, nine genes related to structure and 32 hypothetical genes (Figure 1a). The BREN6 genome possesses two genes, encoding a Cas4‐domain endonuclease and a MazG‐like pyrophosphatase, which can enable evasion of host CRISPR‐Cas and abortive infection host defence systems, respectively (Ho et al. 2023; Hooton and Connerton 2015). No genes related to lysogeny, antimicrobial resistance (AMR) or bacterial virulence were identified within the BREN6 genome.

Notably, phage BREN6 showed no significant genomic similarity to any pre‐existing phage genomes in the Virus Metadata Resource (VMR) database and is likely to belong to a new genus and species. Proteomic analysis of BREN6, using VipTree, showed that BREN6 has some similarity to phages with siphovirus morphology that infect other Proteobacteria species (Figure 1b), such as the *Rhodobacter capsulatus* phages RcTitan (Genomic similarity scores‐SG = 0.2483) and RcSpartan (SG = 0.2486); *Burkholderia cenocepacia* phage KL1 (SG = 0.3416); and *Xanthomonas translucens* phages Langgruindblatt1/2 (SG = 0.2501) and Pfeifenkraut (SG = 0.243) (Bollivar et al. 2016; Erdrich et al. 2022; Lynch et al. 2012).

All five GIB phages isolated also had 100% nucleotide identity, and so phage GIB1 was chosen as a representative of this set of phages. The genome of GIB1, available under the GenBank accession PZ136971, is 75,744 bp long and has a GC content of 43.88%. It contains two tRNAs and 99 predicted genes, of which 25 were related to structure, three to host lysis and 10 to DNA binding and packaging. No genes related to lysogeny, AMR or bacterial virulence were identified within its genome. GIB1 also possesses a lysis inhibitor cassette composed of *rIIA* and *rIIB* lysis inhibitor genes, which may enable a delay in host lysis, leading to an increased burst size (Lerdsittikul et al. 2022). A gene coding for a Mu Gam‐like end protection protein is also present in the GIB1 genome, which is suggested to protect phage genome termini from exonucleases during viral DNA packaging (Bhattacharyya et al. 2018). GIB1 has two tRNAs, which have various potential functions in phages, including evasion of the host defence system, or compensation for the difference in codon usage between the phage and its host (Lomeli‐Ortega and Balcázar 2024). GIB1 also possesses a DNA adenine methyltransferase (DAM), which has been shown to protect coliphages T4 and P1 from host restriction modification systems during infection (Murphy et al. 2013). Another gene of interest encoded a Hoc‐like head decoration. Hoc is a highly antigenic outer capsid protein that likely aids phage binding to different surfaces, thereby enabling its survival (Sathaliyawala et al. 2010). Finally, GIB1 possesses a gene for a lipoprotein that shares homology with the 123 stomatin/prohibitin/flotillin/HflK/HflC (SPFH) domain‐containing proteins. The SPFH domain is linked to lipid rafts, which contribute to bacterial membrane fluidity and protein trafficking; however, the reason it is found within a phage genome remains unclear (Browman et al. 2007). Even so, given its predicted function, we hypothesise that it may be involved in phage adsorption or entry of phage DNA into the cell.

Taxonomic analysis by taxmyPHAGE showed that phage GIB1 was classified within the family *Schitoviridae* and genus *Efbeekayvirus*. The genome size and genomic presence of three RNA polymerases of GIB1 are typical of the *Schitoviridae* family, which is composed of N4‐like phages with a podovirus morphology (Wittmann et al. 2020). GIB1 has ~91% percentage identity with several phages infecting *Klebsiella pneumoniae* , including FBKp27, a lytic phage isolated from sewage (Figure 1c) (Bonilla et al. 2021). Proteomic analysis using VipTree showed that alongside FBKp27 (SG = 0.6815), GIB1 has similarities to other N4‐like phages, such as *Acinetobacter baumanii* phage Presley (SG = 0.156), *Kosakonia cowanii* phage Kc283 (SG = 0.2919) and *Pectobacterium versatile* phage Possum (SG = 0.2823) (Figure 1d) (Farmer et al. 2013; Fouts et al. 2013; Lukianova et al. 2022; Petrzik et al. 2021).

#### 3.2.2 BREN and GIB Phages Possess *Caudoviricetes* Morphology

**FIGURE 2:**
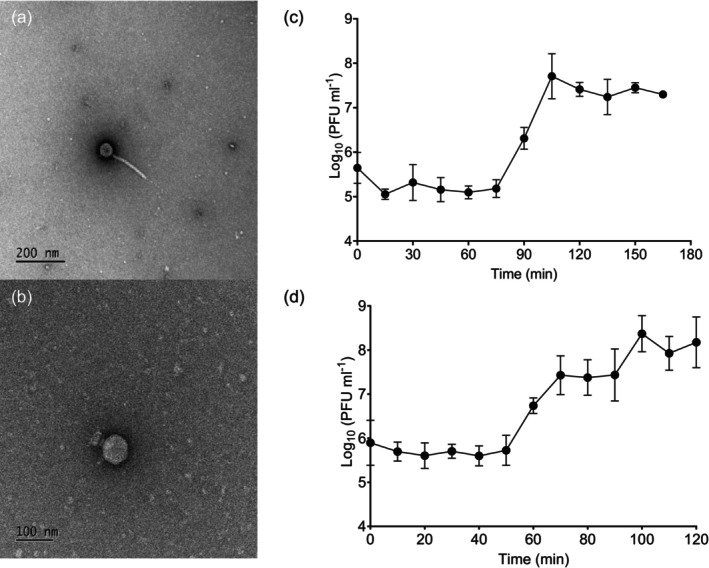
Characterisation of phages BREN6 and GIB1. Transmission electron microscopy images are displayed showing the siphovirus and podovirus morphology of phages BREN6 (a) and GIB1 (b), respectively. One‐step growth curves of phage BREN6 against *Bg* FRB 141^T^ and phage GIB1 against *Gq* FRB 124 are shown in (c) and (d), respectively. Each point represents the mean of three replicates, and bars represent standard error.

Transmission electron microscopy was performed on the two representative phages, BREN6 and GIB1, to determine their physical morphology. Both phages belong to the class *Caudoviricetes*, as they possess icosahedral capsids and tails (Turner et al. 2023). BREN6 has a long tail averaging 163.7 nm in length and an average capsid width of 54.1 nm, exhibiting siphovirus morphology (Figure 2a). It also possesses a single long central tail spike of ~95.6 nm. GIB1 showed a podovirus morphology (Figure 2b), with an average capsid head width of 66.7 nm and tail length of 33.6 nm.

#### 3.2.3 One‐Step Growth Curves Revealed Typical Phage Growth Kinetics

One‐step growth curves were performed to determine the growth kinetics of the phages. The latent period of BREN6 was 75 min, and its rise period lasted 30 min, with a burst size of an average of 101 virions per infected cell (Figure 2c). The latent and rise periods of GIB1 were approximately 50 and 20 min, respectively (Figure 2d). Its burst size was an average of 58 virions per infected bacterial cell.

#### 3.2.4 BREN and GIB Phages Have Narrow Host Ranges

BREN and GIB phages were tested against several different strains of their hosts, as well as closely related species and other species found within the AOD pathobiome, to determine the extent of their host range. BREN6 could lyse the type strain that was used for the initial isolation (*Bg* FRB 141^T^, isolated from Outwoods in Leicestershire) and over half (8/15) of the *Bg* strains tested did not lyse any species other than *Brenneria goodwinii* (Table S2). Similarly, GIB1 could lyse four of the 13 strains tested, and could not lyse any species other than *Gibbsiella quercinecans* (Table S2). Interestingly, BREN6 could lyse the *Bg* strains isolated from the same location as itself, Wyre Forest, but could not lyse *Bg* strains isolated from Norbury or from a range of other UK woodlands. Conversely, GIB1 could not lyse the *Gq* strain isolated from Norbury Park. These results suggest that both phages have a narrow host range limited to a small set of species‐specific strains.

### 3.3 The Genomes of Various *Bg* and *Gq* Strains Possessed Multiple Phage Defence Systems

The host range assay showed that there were several strains of *Bg* and *Gq* that were naturally resistant to phages BREN6 and GIB1, respectively. One way that bacteria can prevent phage infection is by deploying phage defence systems. Defence systems that could be associated with natural resistance to BREN6 and GIB1 were, therefore, identified in the genomes of different susceptible and resistant *Bg* and *Gq* strains using PADLOC. While the genomes of *Bg* FRB 141^T^ (GCA_002291445.1), *Bg* FRB 171 (GCA_003666145.1), *Bg* FRB 186 (GCA_022361495.1) and *Gq* FRB 97^T^ (GCF_002291425.1) had previously been sequenced, strains *Bg* DI 16a, *Bg* BH 4/25a, *Gq* FRB 124, *Gq* HTL 7.16 and *Gq* Kew 122 were sequenced in this study via hybrid sequencing in order to perform an analysis of their defence systems and sequencing data are available under BioProject number PRJNA1347629.

A single phage defence candidate (PDC‐S07), which encodes the 8‐oxo‐dGTP diphosphatase MutT, was detected in the genomes of all *Bg* and *Gq* strains examined. Eight defence systems were detected within *Bg* FRB 141^T^, and 10 systems were detected in FRB 171 and FRB 186 (strains susceptible to BREN6; Table S3). On the other hand, 12 systems were detected in *Bg* DI16a and *Bg* BH4/25a (strains resistant to BREN6). Notably, *Bg* DI16a and *Bg* BH4/25a both possessed type I restriction modification and type II CRISPR‐cas systems, which were absent within phage‐susceptible *Bg* FRB 141^T^, FRB 171 and FRB 186. Three of the 54 spacer sequences identified within the *Bg* DI 16a CRISPR cassette had high homology to *Salmonella* phage L6jm, *Serratia nevei* plasmid CUVET18‐1371, and *Klebsiella pneumoniae* plasmid pKPST617, respectively (Leelapsawas et al. 2024). Six of the 107 spacer sequences within *Bg* BH4/25a were identical to sequences from the BREN6 genome, namely, genes encoding four different hypothetical proteins, a DNA primase and a structural protein.

The genomes of *Gq* FRB 97^T^ and FRB 124 (susceptible to GIB1), and HTL 7.16 and Kew 122 (resistant to GIB1), contained genes for 12, 23, 16 and 14 phage defence systems, respectively (Table S3). All four strains possessed a type I CRISPR‐cas system, a cyclic‐oligonucleotide‐based anti‐phage signalling system (CBASS) and a restriction modification system. None of the CRISPR arrays within any of the strains were similar to phage or plasmid sequences within the BLASTn database or to GIB1. The two resistant strains (HTL 7.16 and Kew 122) possessed a Gabija defence system, which was not present in the susceptible strains (FRB 97^T^ and FRB 124).

Complementary to the previous analysis, PHASTEST was used to identify the location of any prophages, which can often be associated with defence systems (Table S4). One to three intact prophages were present in the genomes of different *Bg* strains, but none co‐located with defence systems. Only one *Gq* strain, HTL 7.16, possessed an intact prophage, which was also not associated with a defence system.

### 3.4 BREN and GIB Phages Favour Storage at Low Temperatures

**FIGURE 3:**
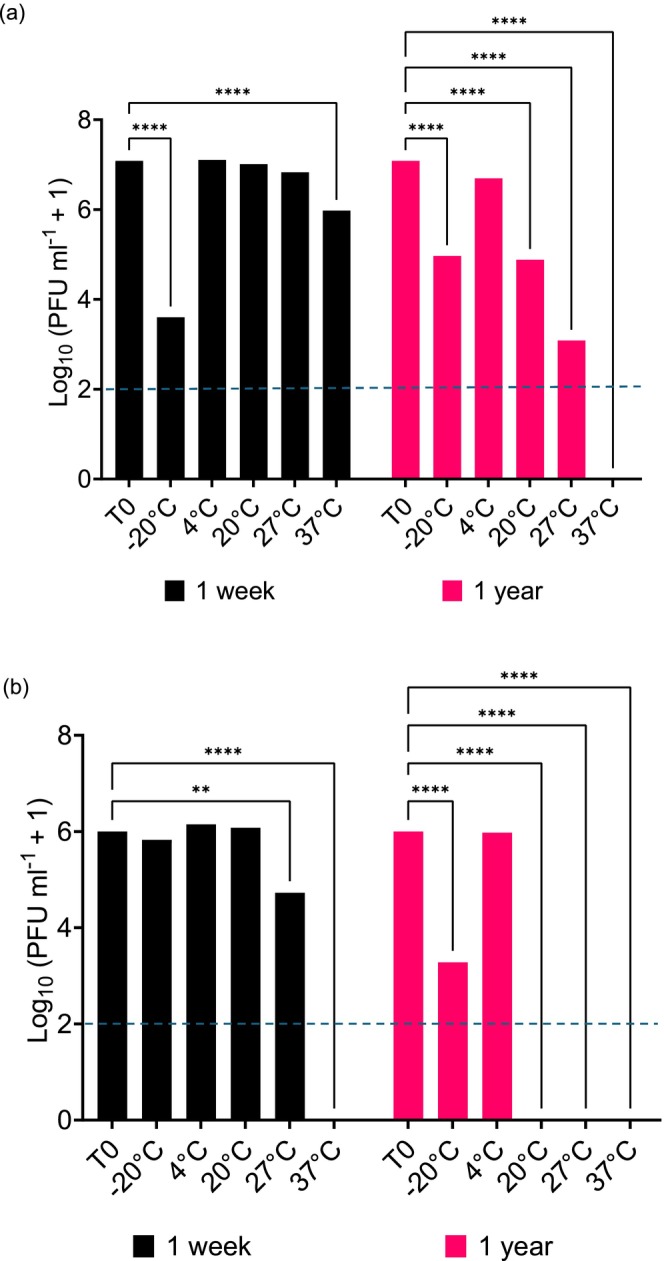
Survival characteristics of phages stored at different temperatures. Titres of phages BREN6 (a) and GIB1 (b) after storage in PBS at different temperatures over 1 week and 1 year. The *y*‐axis represents log_10_(*x* + 1) values of the plaque forming units (PFU) per millilitre of each phage population. Each bar represents the mean of three phage replicate stocks. T0 represents the starting titre of the phages, and each line represents the significant difference to T0 following GLMM and Tukey's HSD tests. Significance relates to the following symbols: **p < 0.01; ****p < 0.0001. The limit of quantification (LOQ) is indicated by the blue dotted line (100 PFU mL^−1^).

A temperature assay was performed to determine the stability of the phages at various temperatures and to identify their optimal storage temperature. Phages suspended in PBS were stored at temperatures ranging from −20°C to 37°C for one week and one year. A GLMM with a Poisson distribution and Tukey's HSD tests were performed to compare each phage's final titre to their starting titre (T0). The complete results of these tests are recorded in Table S5. There was a significant impact of time (*χ* ^2^ = 511.53, *p*‐value < 0.0001) and its interaction with temperature (*χ* ^2^ = 3004.04, *p*‐value < 0.0001) on BREN6 titre during storage, but not of temperature alone (*χ* ^2^ = 0, *p*‐value = 1). After 1 week, BREN6 stocks had significantly reduced in titre at −20°C and 37°C (−20°C: estimate = −3.483, 95% CI [−3.1733, −3.7927], *p*‐value < 0.0001; 37°C: estimate = −1.1096, 95% CI [−1.4192, −0.7999], *p*‐value < 0.0001; Figure 3a). After 1 year, BREN6 stocks stored at all temperatures except 4°C were significantly reduced.

There was also a significant impact of time (*χ* ^2^ = 115.11, *p*‐value < 0.0001) and its interaction with temperature (*χ* ^2^ = 626.32, *p*‐value < 0.0001) on GIB1 titre, but no significant impact of temperature alone (*χ* ^2^ = 0, *p*‐value = 1). After one week, GIB1 stocks could not be detected at all at 37°C compared to T0 (estimate = −5.667, 95% CI [−6.398, −4.935], *p*‐value < 0.0001) and had significantly declined at 27°C (estimate = −1.111, 95% CI [−1.842, −0.78], *p*‐value = 0.0011); however, they had not dropped in titre at −20°C, 4°C or 20°C. After one year, GIB1 stocks stored at 4°C and −20°C could only be detected (Figure 3b). These results suggest that the optimal storage temperature for maintenance of titre of both BREN and GIB phages is 4°C. These data may also suggest that sampling strategies for phage isolation from environmental samples are influenced by seasonality and are best timed for winter.

### 3.5 Killing Curve Assays Showed Each Phage Effectively Reduced Their Host's Growth

Killing curve assays were performed over 48 h at three different MOIs to determine the ability of the phages in preventing the population growth of their respective hosts and to identify the proportion of phages that was most effective. The absorbance at 600 nm (A600) at 12, 24, 36 and 48 h of each killing curve was measured for each bacterium‐phage pair at each MOI. A GLMM with a Gamma distribution and Tukey's HSD tests were used to determine the fixed effects of time and MOI on A600, with population replicate used as a random effect. All statistical results are recorded in Table S6.

**FIGURE 4:**
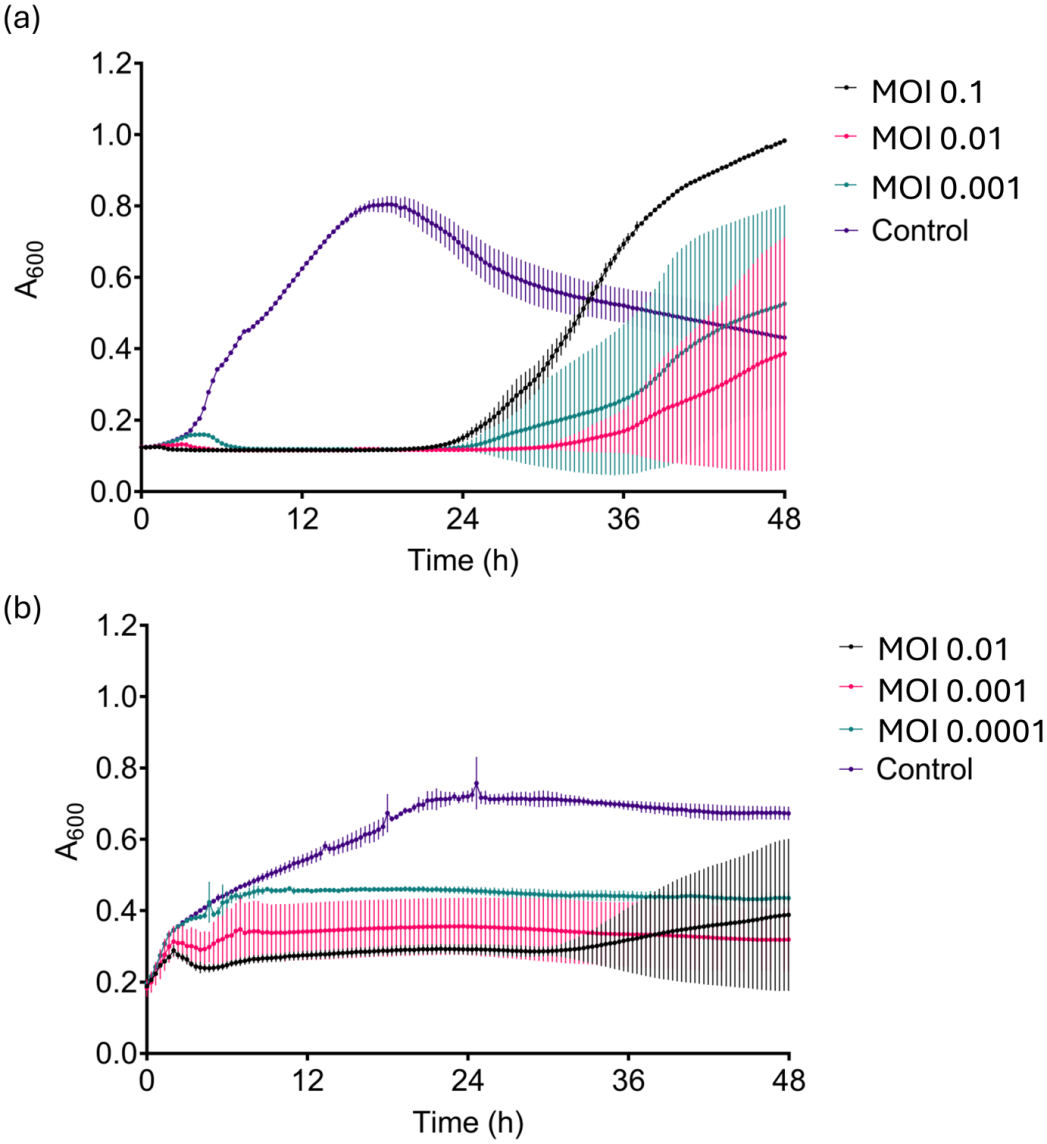
Phages BREN6 and GIB1 kill their host bacterium, but phage‐resistance can occur. BREN6 was incubated with its host *Brenneria goodwinii* FRB 141^T^ (a), and GIB1 with its host *Gibbsiella quercinecans* FRB 124 (b). Different proportions of phages to bacterial cells (MOI) were tested (MOI 1 to 10^−7^) for each phage‐bacterium pair. Controls contained the bacterial population with PBS instead of phages. Absorbance at 600 nm (A600) was recorded every 20 min for 48 h. Each curve represents the mean and standard deviation of three replicates.

There was a significant effect of phage treatment MOI (*χ* ^2^ = 78.194, *p*‐value < 0.0001) and its interaction with time (*χ* ^2^ = 101.208, *p*‐value < 0.0001) on the A600 of *Bg* populations, but no significant effect of time only (*χ* ^2^ = 2.534, *p*‐value = 0.4692; Figure 4a). The A600 of *Bg* populations treated with BREN6 phages at all MOIs was significantly lower than the untreated control *Bg* populations at 12 and 24 h, but there were no differences within MOI treatments (Table S6). However, by the end of the assay (48 h), the A600 of *Bg* populations treated with phages at MOI 0.01 and MOI 0.001 was not different to that of the control, whereas populations treated with the highest MOI (0.1) were significantly higher than the control (estimate = 0.6288, 95% CI [0.0904, 0.78], *p*‐value = 0.0315). Phage‐treated *Bg* populations saw a sudden steep increase after approximately 24 h of incubation, which is likely caused by the emergence of phage resistance. It is possible that the phage treatment with the highest MOI (0.1) inflicted the highest selection pressure for phage‐resistance mutations and therefore caused the most significant increase in later bacterial growth.

The MOI of GIB1 treatment had a significant effect on the A600 of *Gq* populations (*χ* ^2^ = 30.5957, *p*‐value < 0.0001; Figure 4b), but there was no significant effect of time (*χ* ^2^ = 5.2525, *p*‐value = 0.1542) or the interaction between time and MOI (*χ* ^2^ = 10.4985, *p*‐value = 0.3117). Notably, the A600 of all phage‐treated *Gq* populations was significantly lower than that of the untreated control populations at all time points (Table S6). There was no significant difference between the A600 of populations treated with an MOI of 0.01 and 0.001 at any time point. However, populations treated with an MOI of 0.01 saw a slight increase towards the end of the experiment, suggestive of phage‐resistance emergence. Populations treated with an MOI of 0.0001 were significantly higher than those treated with an MOI of 0.01 or 0.001 at 12, 24 and 36 h, suggesting that this lower MOI was less effective in preventing *Gq* population growth. These results demonstrate that BREN6 and GIB1 are effective in preventing the growth of their host populations for at least 24 h, but that phage‐resistance emergence was accelerated at higher MOIs.

### 3.6 Phages Had a Minimal Impact on Bacterial Populations *In Planta*

The interaction between a phage and its host *in planta* can be very different in comparison to in vitro (Hernandez and Koskella 2019). To determine if the phages could reduce a community of *Bg* and *Gq* within tree stem tissues, both bacteria and phage types were co‐inoculated into wounds on the stems of large oak saplings, and population densities were quantified at 0‐, 5‐, 10‐ and 30‐days post‐inoculation (dpi). Data were assessed using GLMM and Tukey post hoc tests, and full statistical results are provided in Tables S7a and S7b.

**FIGURE 5:**
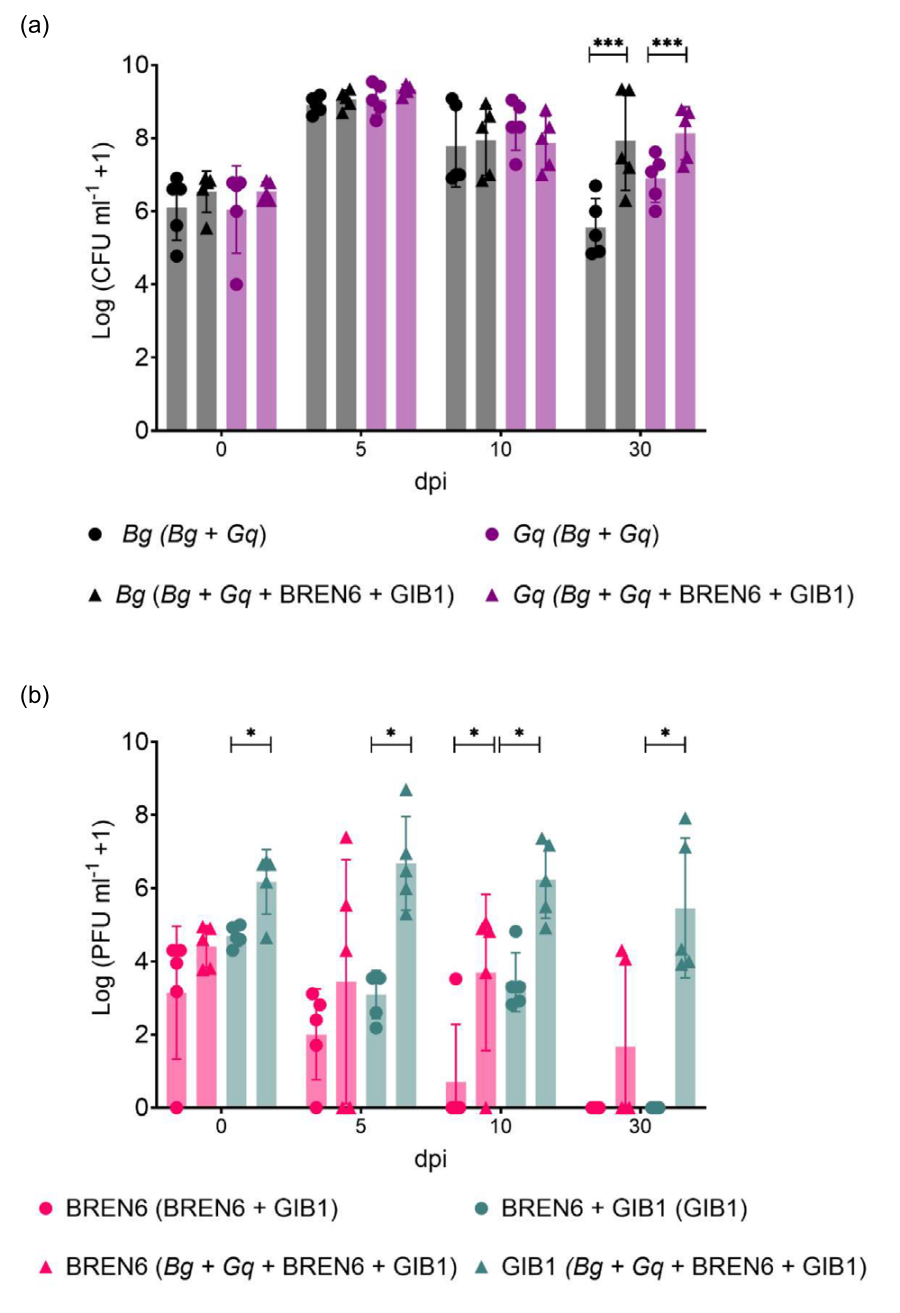
Phage application to infected trees causes small increases in pathogen populations, but phage populations diminish. Population densities of bacteria *Bg* and *Gq*, and phages BREN6 and GIB1, were measured over 30 days within stem lesions on *Q. robur* saplings. (a) Densities of *Bg* and *Gq* are shown over time as a community with (*Bg* + *Gq* + BREN6 + GIB1) and without (*Bg* + *Gq*) the presence of phages. (b) Densities of BREN6 and GIB1 are shown with (*Bg* + *Gq* + BREN6 + GIB1) and without (BREN6 + GIB1) the presence of their hosts. Colony and plaque forming units (CFU/PFU) per millilitre of oak homogenate were transformed using log_10_(*x* + 1) for bacteria and phages, respectively. Each point represents the mean of three technical replicate measurements of bacterial or phage density from one wound sample, and bars represent the mean and standard deviation of all samples (*N* = 5). Bacteria and phage densities were compared dependent on phage or control treatment, and between sampling time points, using a GLMM and Tukey's HSD tests, with statistical significance indicated by star symbols (*p < 0.05; ***p < 0.001). The limit of quantification (LOQ) for phages is indicated by the blue dotted line (100 PFU mL^−1^).

There was a significant effect of time, and a significant interaction between time and bacterial and phage combination on *Bg* (time: *χ* ^2^ = 87.9004, *p*‐value = < 0.0001; time × combination: *χ* ^2^ = 35.6833, *p* < 0.0001; Figure 5a) and *Gq* (time: *χ* ^2^ = 49.3072, *p* < 0.0001; time × combination: *χ* ^2^ = 11.2966, *p* = 0.0102; Figure 5a) populations. *Bg* and *Gq* population densities in wounds treated with both the control (‘*Bg* + *Gq*’) and the phage treatment (‘*Bg* + *Gq* + BREN6 + GIB1’) rose significantly from 0 to 5 dpi (Table S7a), suggesting that all bacterial populations grew *in planta* following inoculation, regardless of phage presence. There was no significant difference in *Bg* or *Gq* populations treated with phages compared to those without at 0, 5 or 10 dpi. Interestingly, at 30 dpi, *Bg* and *Gq* populations that had been treated with phages were significantly higher than those from control‐treated wounds (*Bg*: estimated means = 0.3536, 95% CI [0.2200, 0.4871], *p* < 0.0001; *Gq*: estimated mean = 0.1655, 95% CI [0.0521, 0.279], *p* = 0.0042). This suggests that, instead of reducing bacterial populations as seen in in vitro, BREN6 and GIB1 phages *in planta* have little impact on their respective hosts and may in fact result in larger bacterial population sizes.

There was no statistically significant impact of time (BREN6: *χ* ^2^ = 6.3131, *p* = 0.09733; GIB1: *χ* ^2^ = 2.3359, *p* = 0.2720), combination (BREN6: *χ* ^2^ = 0.417, *p* = 0.5184; GIB1: *χ* ^2^ = 0.9931, *p* = 0.319) or their interaction (BREN6: *χ* ^2^ = 3.9040, *p* = 0.2720; GIB1: *χ* ^2^ = 1.9886, *p* = 0.5748) on the BREN6 or GIB1 population densities (Figure 5b; Table S7b). When phages were applied to stem wounds without their hosts (‘BREN6 + GIB1’), phage populations generally declined over time, though this observation was not significant. Neither phage type could be detected at 30 dpi when inoculated without their hosts. When phages were applied alongside their hosts (‘*Bg* + *Gq* + BREN6 + GIB1’), GIB1 populations were maintained at similar densities over each time point. However, some BREN6 populations did go extinct at 5, 10 and 30 dpi, suggesting that the survival of these phages *in planta* is unstable and dependent on the presence of their host.

To determine if phage resistance arose *in planta* in *Bg* and *Gq* as was observed in vitro, *Bg* and *Gq* colonies from 30 dpi were isolated and assessed for phage resistance via a killing curve assay using their respective ancestral phages. The growth of all bacterial isolates tested was reduced by their respective phage type, indicating that *in planta* bacterial populations remained susceptible to phages at 30 dpi (Figure S3).

### 3.7 There Was No Impact of Phages on Either Single or Two Species Bacterial Communities *In Planta*

The previous experiment explored the dynamics of *Bg*, *Gq* and their phages in oak stem wounds over time when *Bg* and *Gq* were co‐inoculated together with and without both phages as a community, as naturally occurring oak bleeding cankers often contain multiple pathogens. However, to determine if the ability of one phage in lysing their host was affected by the presence of the other phage‐bacteria pair, phages were tested against *Bg* and *Gq* both singularly and in a community within oak stem wounds. Bacteria and phages were quantified immediately after inoculation, and after 14 dpi, a time known to allow bacterial establishment and when bacteria and phage could still be isolated from the trees. As the data were non‐parametric, an ART ANOVA and Tukey post hoc tests were used to determine if population densities of *Bg*, *Gq*, BREN6 and GIB1 differed depending on timepoint and the bacteria‐phage combination applied; all statistical results are supplied in Tables S8a and S8b.

**FIGURE 6:**
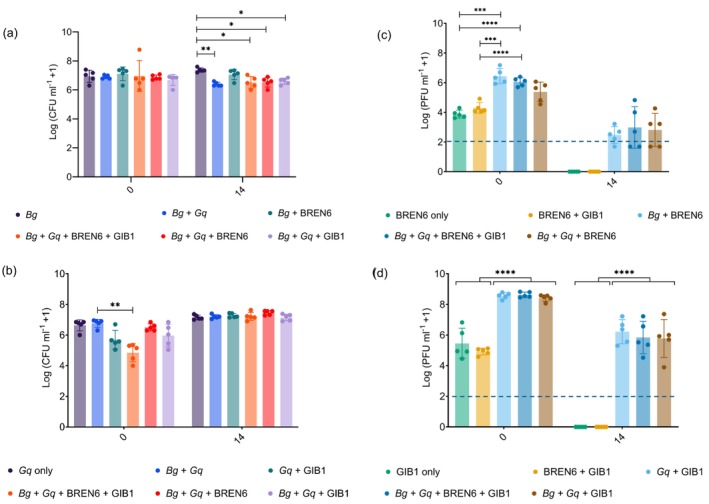
Phages do not have an adverse effect on single or dual species bacterial communities. Population densities of bacteria *Bg* and *Gq*, and phages BREN6 and GIB1, within oak stem tissue when applied in monoculture (‘*Bg* only’; ‘*Bg* + BREN6’; ‘*Gq* only’; ‘*Gq* + GIB1’) or community combinations (‘*Bg* + *Gq*’; ‘*Bg* + *Gq* + BREN6’; ‘*Bg* + *Gq* + GIB1’; ‘*Bg* + *Gq* + BREN6 + GIB1’) were measured, immediately after inoculation (0 dpi) and 14 days post‐inoculation (14 dpi). Densities of *Bg*, *Gq*, BREN6 and GIB1, where present in these combinations, are shown in (a), (b), (c) and (d), respectively. Colony and plaque forming units (CFU/PFU) per millilitre of oak homogenate were transformed using log_10_(*x* + 1) for bacteria and phages, respectively. Each point represents the mean of three technical replicates of bacterial or phage density from one wound sample, and bars represent the mean and standard deviation of all samples (*N* = 5). Bacteria and phage densities were compared dependent on combination and sampling time point, using an ART ANOVA and Tukey's HSD tests, with statistical significance indicated by star symbols (*p < 0.05; **p < 0.01; ***p < 0.001; ****p < 0.0001). The limit of quantification (LOQ) for phages is indicated by the blue dotted line (100 PFU mL^−1^).

There was a significant impact of time (*Bg*: *χ* ^2^ = 5.138, *p* = 0.03; *Gq*: *χ* ^2^ = 133.648, *p* < 0.0001), combination (*Bg*: *χ* ^2^ = 4.262, *p* = 0.0003; *Gq*: *χ* ^2^ = 10.162, *p* < 0.0001) and their interaction (*Bg*: *χ* ^2^ = 3.251, *p* = 0.013; *Gq*: *χ* ^2^ = 11.544, *p* < 0.0001) on *Bg* and *Gq* populations. At 0 dpi, there was no significant difference in any *Bg* population densities dependent on combination (Figure 6a). At 14 dpi, there was no difference in the population of *Bg* treated with BREN6 compared to *Bg* alone. However, *Bg* population densities in ‘*Bg* only’ were significantly higher than that of all populations that also contained *Gq* (Table S8a). This may indicate that while *Bg* populations are not significantly affected by BREN6 *in planta*, their growth is constrained by the presence of *Gq*.

All GIB1‐treated *Gq* populations (i.e., those within ‘*Gq* + GIB1’; ‘*Bg* + *Gq* + GIB1’; ‘*Bg* + *Gq* + BREN6 + GIB1’) at 0 dpi were lower than those that did not contain GIB1 (‘*Gq* only’, ‘*Bg* + *Gq*’, ‘*Bg* + *Gq* + BREN6’). However, this difference was only statistically significant between ‘*Bg* + *Gq*’ and ‘*Bg* + *Gq* + BREN6 + GIB1’ (rank mean = 21.6, 95% CI = [12.4664, 30.7336], *p* = 0.0015; Figure 6b). At 14 dpi, there was no difference in *Gq* population densities between any combination, suggesting that GIB1‐treated *Gq* populations were no longer affected by GIB1 at this later time point and that *Bg* did not impact *Gq* populations.

There was also a significant impact of time (BREN6: *χ* ^2^ = 130.2518, *p* < 0.0001; GIB1: *χ* ^2^ = 126.58, *p* < 0.0001) and combination (BREN6: *χ* ^2^ = 8.0647, *p* < 0.0001; GIB1: *χ* ^2^ = 30.69, *p* < 0.0001) on BREN6 and GIB1 populations. However, there was only a significant interaction between time and combination for GIB1 (BREN6: *χ* ^2^ = 1.1508, *p* = 0.3469; GIB1: *χ* ^2^ = 18.247, *p* < 0.0001). BREN6 populations at 0 dpi that were in combinations that contained their host *Bg* (i.e., ‘*Bg* + BREN6’, ‘*Bg* + *Gq* + BREN6’, ‘*Bg* + *Gq*’, ‘*Bg* + *Gq* + BREN6 + GIB1’) were higher than those in combinations that did not (i.e., ‘BREN6 only’, ‘BREN6 + GIB1’) at 0 and 14 dpi, but this difference was only significant at 0 dpi (Table S8b; Figure 6c). Likewise, GIB1 populations in combinations that contained *Gq* were higher than those that did not contain *Gq*, and this difference was statistically significant at 0 and 14 dpi (Table S8b; Figure 6d). These results reinforce the observation that BREN6 and GIB1 survive better *in planta* when alongside their hosts and show that the presence of another phage‐bacteria pair does not impact the survival of either phage.

## 4 Discussion

*Brenneria goodwinii* (*Bg*) and *Gibbsiella quercinecans* (*Gq*) are Gram‐negative bacteria that cause bleeding cankers on the stems of oak trees suffering from acute oak decline (AOD). In this study, the first lytic phages that infect these two species have been isolated and characterised. The *Bg*‐infecting phage, BREN6, and the *Gq*‐infecting phage, GIB1, were isolated from oak cankers within two woodlands impacted by AOD. Initial investigation of the phages involved assessing their potential as biocontrol candidates. Both phages could effectively reduce their host populations in vitro; could not lyse any bacterial species tested other than the target hosts; and did not possess any genes related to AMR, bacterial virulence, or AMR.

BREN6 and GIB1 were then tested for their ability to reduce their host populations *in planta*. However, when applied against their bacterial hosts within oak tree stem wounds, the phages did not significantly reduce the populations of these bacteria. It is not uncommon for phages to be less effective in reducing their bacterial host populations *in planta* (Balogh et al. 2018; Saccardi et al. 1993). One possible reason for this is that the spatial heterogeneity of the oak stem wound created protective spatial niches for bacteria, which reduced bacteria‐phage encounters (Brockhurst et al. 2006), resulting in a lower rate of phage lysis. Secondly, as phage movement occurs via passive diffusion or hitchhiking alongside their host, prevention of phage movement towards bacterial populations may have also occurred due to the spatial heterogeneity of the tissue, particularly necrotic tissue that may have blocked phage access to the phloem and xylem. Thirdly, phage presence may have influenced quorum sensing within *Bg* and *Gq* populations, which can result in altered bacterial behaviour to evade phage predation, such as activation of phage defence systems or biofilm production (León‐Félix and Villicaña 2021; Simmons et al. 2020). Another possibility is that the harsher conditions of the plant environment, such as lower moisture, suboptimal temperatures and the presence of UV irradiation, reduced the phages' lytic abilities. This experiment was carried out on oak saplings grown within a controlled environment glasshouse, at which the temperature was maintained between 16°C and 22°C. As observed from the phage storage experiment, the phages survived best at the lower temperature of 4°C, and thus this experimental approach, optimised for the seasonal timing when cankers occur, may have disfavoured phage survival. Additionally, as oak stem tissue and bark are rich in antimicrobial metabolites such as tannins (Vinchira‐Villarraga et al. 2025), it is also possible that the tissue may have had an antiviral impact on the phage, reducing their lytic ability (Huang et al. 2024). The latter two reasons are further supported by the observation that populations of BREN6 and GIB1 applied without the presence of their hosts reduced significantly over time and could not be detected after 30 days, suggesting that they struggled to persist in the oak stem environment.

The development of phage resistance can also reduce the efficacy of phage treatments, yet no phage resistance was detected in *Bg* and *Gq* colonies isolated from trees 30 days post phage application. While phage resistance can readily occur in vitro due to point mutations to genes encoding phage receptors, such as lipopolysaccharide and flagella (Oechslin 2018), it is less commonly seen *in planta*. Phage‐resistance mutations can incur fitness defects, and therefore phage resistance can be costly within the harsher plant environment and thus be constrained from dominating within bacterial populations (Hernandez and Koskella 2019). Several other studies have also been unable to isolate phage‐resistant bacterial populations from plant systems (Hernandez and Koskella 2019; Papp‐Rupar et al. 2025; Ramírez et al. 2020). For example, Das et al. (2015) could not isolate resistant *Xylella fastidiosa* populations from grapevines treated with a phage cocktail after 12 weeks of infection.

To investigate if BREN6 and GIB1 phages had an impact on the interaction between *Bg* and *Gq* *in planta*, different combinations containing each bacterium‐phage pair singularly or together in a community were applied to oak tree saplings. While GIB1 did reduce populations of *Gq* at 0 dpi in both single (‘*Gq* + GIB1’) and mixed (‘*Bg* + *Gq* + BREN6 + GIB1’) species communities, it did not at 14 dpi, suggesting that the impact of GIB1 on *Gq* populations was short‐lived. Alternatively, *Bg* populations were significantly lower at 14 dpi when applied alongside *Gq*, but were not significantly impacted by BREN6, suggesting that the presence of *Gq* rather than BREN6 limits the growth of *Bg* populations. These findings show that the application of phages does not alter population dynamics between *Bg* and *Gq* in *in planta* communities. This was an intriguing result, as phages have been shown to significantly alter multispecies communities. For example, cellular debris from a lysed phage‐susceptible bacterial species can provide nutrients for a non‐susceptible bacterial species, enabling its increased growth (Fazzino et al. 2020). Alternatively, the reduction of a dominant bacterial species by phages can facilitate an increase in less competitive species, leading to an increase in species richness within a community (Alseth et al. 2024). In this study system, it is possible to hypothesise that *Bg* and *Gq* occupy different spatial or nutrient niches within the oak stem, or cannot consume each other's bacterial debris, and therefore the predation by phage of one bacterial species does not impact the other.

An unexpected result was that phage‐treated bacterial populations in two trees were significantly higher than control‐treated trees at 30 dpi. One possible explanation for this is that phage predation pressure resulted in increased bacterial dispersion which enabled *Bg* and *Gq* to tap into new resource niches, or selected for a bacterial phenotype with a higher growth rate. *Bg* and *Gq* isolates from the *in planta* experiments should be investigated to determine if the bacterial phenotypes altered over time to become better adapted to the tree tissue environment. Moreover, while we do not know whether *Bg* and *Gq* can consume each other's cell debris, phage lysis may have enabled increased bacterial population growth by allowing each species to consume their own species' cell debris.

However, these results support the hypothesis that *Bg* and *Gq* do initially interact competitively (Brady et al. 2022). Co‐occurring bacteria can compete for resources such as space and nutrients. This competition can occur by harming a competitor (direct competition), such as by the production of antimicrobial compounds or activation of a type 6 secretion system; or by better consuming shared resources (indirect competition), such as by activating genes associated with motility, biofilm production or siderophore production (Ghoul and Mitri 2016; Hibbing et al. 2010). Further research should elucidate which resources *Bg* and *Gq* are competing for and what genes they are expressing that enable them to interact competitively while avoiding phage predation.

Genomic analysis showed that naturally occurring *Bg* and *Gq* strains contain a large range of phage defence systems. Genomes of *Escherichia coli* strains, for example, have on average between five and seven defence systems (Wu et al. 2024), whereas the *Bg* strains investigated in this study had between eight and 13, and *Gq* strains between 11 and 23. Activation of these phage defence systems within *Bg* and *Gq* may have contributed to the lack of phage effectiveness *in planta*. Considering the genomes of BREN6 and GIB1 also contained genes associated with anti‐phage defence, such as those encoding a DNA adenine methyltransferase and a MazG‐like pyrophosphatase, these results may also suggest that, in order to acquire and retain these defence systems, naturally occurring populations of *Bg*, *Gq* and their phages have undergone significant arms race dynamics in their evolutionary past.

One commonly found defence system within the bacterial strains investigated was a CRISPR‐cas type I system. Interestingly, the CRISPR array within *Bg* BH4/25a genome contained multiple spacers homologous to genes within phage BREN6, which suggests that this strain has previously encountered similar phages to BREN6, despite *Bg* BH4/25a and BREN6 being isolated from separate locations and time points. *Bg* DI 16a was also resistant to BREN6, but lacked CRISPR array spacers matching BREN6, suggesting that an alternate system may actively prevent BREN6 infection, or that it carries a receptor mutation preventing phage attachment. Other defence systems identified in *Bg* and *Gq* function by abortive infection, which trigger cell death following detection of phage DNA or adsorption, such as Zorya, Lamassu, PARIS and AbiE (Doron et al. 2018; Dy et al. 2014; Millman et al. 2022; Rousset et al. 2022). Others cause the manipulation or degradation of invading phage nucleic acids, preventing their transcription, such as DNA phosphorothioation systems (encoded by *dnd* genes), Septu, Gabija and AVAST (Antine et al. 2024; Cheng et al. 2021; Gao et al. 2020; Jiang et al. 2024).

This study has shown that while BREN6 and GIB1 phages showed promise in their ability to reduce their respective hosts' populations in vitro, they could not reduce them *in planta*. Instead, the phages and their bacterial hosts appeared to steadily co‐exist within oak stem wounds over 30 days. These findings have important implications for the field application of bacteriophages to treat bacterial plant diseases and suggest the need for more considered application strategies, including repeated, timed or higher‐dose treatments. In view of using phages specifically as biocontrol for oak bleeding cankers associated with AOD, and potentially other tree diseases, future studies should focus on investigating if the phages could be engineered to become more effective *in planta*, such as by use of protective formulations to prevent phage degradation or coevolution within the laboratory to improve their environmental robustness (Born et al. 2015; Kering et al. 2020). Fundamental research into understanding phage‐pathogen dynamics in the context of different trees is also clearly an important future task. To further elucidate the role of phages in AOD canker microbiome dynamics, future research should integrate metagenomic and metaviromic approaches to capture broader microbial community interactions. Such approaches would provide deeper insight into how phages shape bacterial community structure and function within naturally occurring cankers and help clarify their ecological role in disease dynamics.

## Author Contributions

**Emily R. Grace:** investigation, writing – original draft, writing – review and editing, methodology, formal analysis, validation, conceptualization. **Vanja Milenkovic:** methodology, investigation, writing – review and editing, validation. **Katherine G. Hinton:** investigation, writing – review and editing, validation. **Sabrine Dhaouadi:** writing – review and editing, investigation. **Diana Vinchira‐Villarraga:** investigation, writing – review and editing, methodology. **Michael A. Brockhurst:** writing – review and editing. **Mojgan Rabiey:** conceptualization, writing – review and editing, investigation, supervision. **Robert W. Jackson:** conceptualization, funding acquisition, writing – review and editing, supervision, investigation.

## Funding

This work was supported by Jabbs Foundation.

## Conflicts of Interest

The authors declare no conflicts of interest.

## Supporting information


**Figure S1:** Maps of the sampling sites of Norbury Park and Wyre Forest, displaying the location of diseased (i.e., D1, D2 etc.) and healthy (i.e., H1, H2 etc.) trees. Maps were created using OpenStreetMap (OpenStreetMap contributors, 2025), licensed under ODbL, and QGIS (QGIS Development Team 2025).
**Figure S2:** Plaque morphologies of phage BREN6 on a lawn of *Bg* FRB 141^T^ (a) and of phage GIB1 on a lawn of *Gq* FRB 124 (b).
**Figure S3:** The phage susceptibility of *Bg* and *Gq* colonies isolated from oak stem saplings 30 days post infection. *Bg* (a) and *Gq* (b) colonies were tested against phages BREN6 or GIB1 respectively, or a sterile buffer control, via killing curve assay, and the difference between the final absorbance at 600 nm value of the phage‐treated and control‐treated killing curves for each colony was calculated. A difference in A600 close to 0.0 is suggestive of phage resistance, and that over 0.1 suggests phages susceptibility. Colonies were isolated from oak saplings 30 days post infection and were isolated from populations containing either ‘*Bg* + *Gq*’ or ‘*Bg* + *Gq* + BREN6 + GIB1’. Previously generated phage‐resistant colonies and phage‐susceptible colonies (i.e., wild type *Bg* and *Gq*) are shown as controls. Each point represents an individual colony, with 20 colonies tested per combination and 6 control colonies tested per species.
**Table S1:** Bacterial strains used in this study to test the host range of BREN and GIB phages, showing the strain number, the host and location from which they were identified and literature reference if available.
**Table S2:** Host ranges of phages BREN6 and GIB1 are shown. Multiple strains of *Brenneria goodwinii*, *Gibbsiella quercinecans* , other AOD‐associated bacteria and phylogenetically related species were tested for phage susceptibility by spot assay. (+) denotes susceptibility to the phages (plaques arise on bacterial lawn), (−) represents no lysis by the phages (no plaques arise on bacterial lawn), (nt) denotes no test was performed.
**Table S3:** Phage defence systems identified within the genomes of *Bg* and *Gq* strains.
**Table S4:** Prophages identified within the genomes of *Bg* and *Gq* strains. Prophages were identified using PHASTEST (Wishart et al. 2023).
**Table S5:** The outcome of general linear mixed model (GLMM) and Tukey's HSD tests used to determine the impact of time, temperature and replicate number (random effect) on the log transformed number of plaque forming units per millilitre (PFU mL^−1^) formed by BREN6 and GIB1 stocks. Phage stocks were stored in five different temperatures (−20°C, 4°C, 20°C, 27°C and 37°C) and measured after 7 days and 1 year. Post hoc comparisons are shown comparing the original stock PFU mL^−1^ (time 0: T0) to those after 7 days and 1 year.
**Table S6:** The outcome of general linear mixed model (GLMM) and Tukey's HSD tests used to determine the fixed effects of phage treatment MOI and time, and of replicate number as a random effect, on the absorbance at 600 nm (A600) of *Bg* populations treated with BREN6 and *Gq* populations treated with GIB1. Killing curves were performed over 48 h, with time extracted every 12 h. Three‐replicate populations were recorded for each MOI.
**Table S7a:** The outcome of general linear mixed model (GLMM) and Tukey's HSD tests used to determine the impact of time, bacteria and phage combination, and tree on the log transformed number of colony forming units per millilitre (CFU mL^−1^) formed by *in planta* *Bg* and *Gq* populations. Time and combination were fixed effects, whereas tree number was a random effect. Combinations of either bacteria only (‘Bg + Gq’) or bacteria with phages (‘Bg + Gq + BREN6 + GIB1’) were applied to the wounds of oak trees, and measurements were taken immediately after inoculation (0 dpi), then at 5, 10 and 30 dpi. Five individual trees (biological replicates) were tested per treatment, with two technical replicates recorded per tree.
**Table S7b:** The outcome of general linear mixed model (GLMM) and Tukey's HSD tests used to determine the impact of time and bacteria and phage combination on the log transformed number of plaque forming units per millilitre (PFU mL^−1^) formed by *in planta* BREN6 and GIB1 populations. Time and combination were fixed effects, whereas tree number was a random effect. Combinations of either phage only (‘BREN6 + GIB1’) or bacteria with phages (‘Bg + Gq + BREN6 + Gq’) were applied to the wounds of oak trees, and measurements were taken immediately after inoculation (0 dpi), then at 5, 10 and 30 dpi. Five individual trees (biological replicates) were tested per treatment, with two technical replicates recorded per tree.
**Table S8a:** The outcome of aligned rank transformation analysis of variation (ART ANOVA) and Tukey's HSD tests used to determine the impact of time and bacteria and phage combination on the log transformed number of colony forming units per millilitre (CFU mL^−1^) formed by *in planta* *Bg* and *Gq* populations. Combinations of either bacteria only (‘Bg + Gq’) or bacteria with phages (‘Bg + Gq + BREN6 + Gq’) were applied to the wounds of oak trees, and measurements were taken immediately after inoculation (0 dpi), then at 14 dpi. Five individual trees (biological replicates) were tested per treatment, with two technical replicates recorded per tree.
**Table S8b:** The outcome of aligned rank transformation analysis of variation (ART ANOVA) and Tukey's HSD tests used to determine the impact of time and bacteria and phage combination on the log transformed number of plaque forming units per millilitre (CFU mL^−1^) formed by *in planta* BREN6 and GIB1 populations. Combinations of either bacteria only (‘Bg + Gq’) or bacteria with phages (‘Bg + Gq + BREN6 + Gq’) were applied to the wounds of oak trees, and measurements were taken immediately after inoculation (0 dpi), then at 14 dpi. Five individual trees (biological replicates) were tested per treatment, with two technical replicates recorded per tree.

## Data Availability

The datasets supporting the conclusions of this article are included within the article and its Supporting Information.
