# Supplementary material for: Characterisation and In Planta Activity of Bacteriophages That Infect the Key Bacterial Species Associated With Acute Oak Decline
Source: Microb Biotechnol. 2026 Jun 2;19(6):e70394. doi: 10.1111/1751-7915.70394 (PMC13239085; doi:10.1111/1751-7915.70394)
Supplement: Supplementary file 1 — Figure S1: Maps of the sampling sites of Norbury Park and Wyre Forest, displaying the location of diseased (i.e., D1, D2 etc.) and healthy (i.e., H1, H2 etc.) trees. Maps were created using OpenStreetMap (OpenStreetMap contributors, 2025), licensed under ODbL, and QGIS (QGIS Development Team 2025). Figure S2: Plaque morphologies of phage BREN6 on a lawn of Bg FRB 141T (a) and of phage GIB1 on a lawn of Gq FRB 124 (b). Figure S3: The phage susceptibility of Bg and Gq colonies isolated from oak stem saplings 30 days post infection. Bg (a) and Gq (b) colonies were tested against phages BREN6 or GIB1 respectively, or a sterile buffer control, via killing curve assay, and the difference between the final absorbance at 600 nm value of the phage‐treated and control‐treated killing curves for each colony was calculated. A difference in A600 close to 0.0 is suggestive of phage resistance, and that over 0.1 suggests phages susceptibility. Colonies were isolated from oak saplings 30 days post infection and were isolated from populations containing either ‘Bg + Gq’ or ‘Bg + Gq + BREN6 + GIB1’. Previously generated phage‐resistant colonies and phage‐susceptible colonies (i.e., wild type Bg and Gq) are shown as controls. Each point represents an individual colony, with 20 colonies tested per combination and 6 control colonies tested per species. Table S1: Bacterial strains used in this study to test the host range of BREN and GIB phages, showing the strain number, the host and location from which they were identified and literature reference if available. Table S2: Host ranges of phages BREN6 and GIB1 are shown. Multiple strains of Brenneria goodwinii, Gibbsiella quercinecans , other AOD‐associated bacteria and phylogenetically related species were tested for phage susceptibility by spot assay. (+) denotes susceptibility to the phages (plaques arise on bacterial lawn), (−) represents no lysis by the phages (no plaques arise on bacterial lawn), (nt) denotes no test was performed. [file MBT2-19-e70394-s001.docx]

**Supplementary figures and tables**

**Supplementary Table 1.** Bacterial strains used in this study to test the host range of BREN and GIB phages, showing the strain number, the host and location from which they were identified, and literature reference if available.

| Bacteria | Host | Location | Source |
| --- | --- | --- | --- |
| *Gibbsiella quercinecans* FRB 124 | *Quercus* spp. | Outwood, Leicestershire, UK | Brady et al. (2010) |
| *G. quercinecans* FRB 97^T^ | *Quercus* spp. | Hoddeson Park Wood, Hertfordshire, UK | Brady et al. (2010) |
| *G. quercinecans* AT 186 | *Quercus* spp. | Attingham Park, Shropshire, UK | Provided by Dr C. Brady, UWE |
| *G. quercinecans* FOD 9/25 | *Quercus* spp. | Gloucestershire, UK | Provided by Dr C. Brady, UWE |
| *G. quercinecans* HTL 3.3a | *Quercus* spp. | Hatchlands Park, Surrey, UK | Provided by Dr C. Brady, UWE |
| *G. quercinecans* HTL 7.16 | *Quercus* spp. |  | Provided by Dr C. Brady, UWE |
| *G. quercinecans* Kew 122 | *Quercus* spp. | Kew Gardens, London, UK | Provided by Dr C. Brady, UWE |
| *G. quercinecans* Kew 224 | *Quercus* spp. |  | Provided by Dr C. Brady, UWE |
| *G. quercinecans* BH 1656 | *Quercus* spp. | Bovingdon, Hertfordshire, UK | Provided by Dr C. Brady, UWE |
| *G. quercinecans* BH 186 | *Quercus* spp. |  | Provided by Dr C. Brady, UWE |
| *G. quercinecans* WY3 | *Q. robur* | Wyre Forest, Worcestershire, UK | This study |
| *G. quercinecans* WY15 | *Q. robur* |  | This study |
| *G. quercinecans* NOR | *Q. robur* | Norbury Park, Staffordshire, UK | This study |
| *Brenneria goodwinii* FRB 141^T^ | *Quercus* spp. | Outwoods, Leicestershire, UK | Denman et al. (2012) |
| *B. goodwinii* FRB 171 | *Quercus* spp. | Gorse Covert, Leicestershire, UK | Denman et al. (2012) |
| *B. goodwinii* FRB 186 | *Quercus* spp. |  | Denman et al. (2012) |
| *B. goodwinii* FRB 193 | *Quercus* spp. |  | Provided by Dr C. Brady, UWE |
| *B. goodwinii* HTL 1.1b | *Quercus* spp. | Hatchlands Park, Surrey, UK | Provided by Dr C. Brady, UWE |
| *B. goodwinii* SOT 2/28a | *Quercus* spp. | Sotterly Estate, Suffolk, UK | Provided by Dr C. Brady, UWE |
| *B. goodwinii* BH1/28a | *Quercus* spp. | Bovingdon, Hertfordshire, UK | Provided by Dr C. Brady, UWE |
| *B. goodwinii* BH 4/25a | *Quercus* spp. |  | Provided by Dr C. Brady, UWE |
| *B. goodwinii* DI 16a | *Quercus* spp. | Netherlands | Provided by Dr C. Brady, UWE |
| *B. goodwinii* CP 82 | *Quercus* spp. | Clumber Park, Nottinghamshire, UK | Provided by Dr C. Brady, UWE |
| *B. goodwinii* WY14 | *Q. robur* | Wyre Forest, Worcestershire, UK | This study |
| *B. goodwinii* WY19 | *Q. robur* |  | This study |
| *B. goodwinii* J4.2 | *Q. robur* |  | This study |
| *B. goodwinii* J4.3 | *Q. robur* |  | This study |
| *B. goodwinii* Norbury | *Q. robur* | Norbury Park, Staffordshire, UK | This study |
| *B. salicis* 1027 | *Salix* ssp. | UK | Hauben et al. (1998) |
| *B. roseae* sp. *roseae* FRB 222^T^ | *Quercus cerris* | Norfolk, UK | Brady et al. (2014a) |
| *Rahnella victoriana* BRK 225^T^ | *Quercus* spp. | Suffolk, UK | Brady et al. (2014b) |
| *R. victoriana* FRB 189 | *Quercus* spp. | Suffolk, UK | Brady et al. (2014b) |
| *Lonsdalea britannica* 447^T^ | *Quercus robur* | Surrey, UK | Brady et al. (2012) |
| *Raoultella planticola* | *Quercus* spp. | UK | Provided by Dr C. Cunnigham, University of Reading |
| *Pseudomonas syringae* pv. s*yringae* 9097 | *Prunus avium* | Warwickshire, UK | Hulin et al. (2018) |
| *Klebsiella pneumoniae* LM367 | *Homo sapiens* | Birmingham, UK | Provided by Dr A. Snaith, University of Birmingham |

**Supplementary Figure 1.** Maps of the sampling sites of Norbury Park and Wyre Forest, displaying the location of diseased (*i.e.,* D1, D2 etc.) and healthy (*i.e.,* H1, H2 etc.) trees. Maps were created using OpenStreetMap (OpenStreetMap contributors, 2025), licensed under ODbL, and QGIS (QGIS Development Team, 2025).


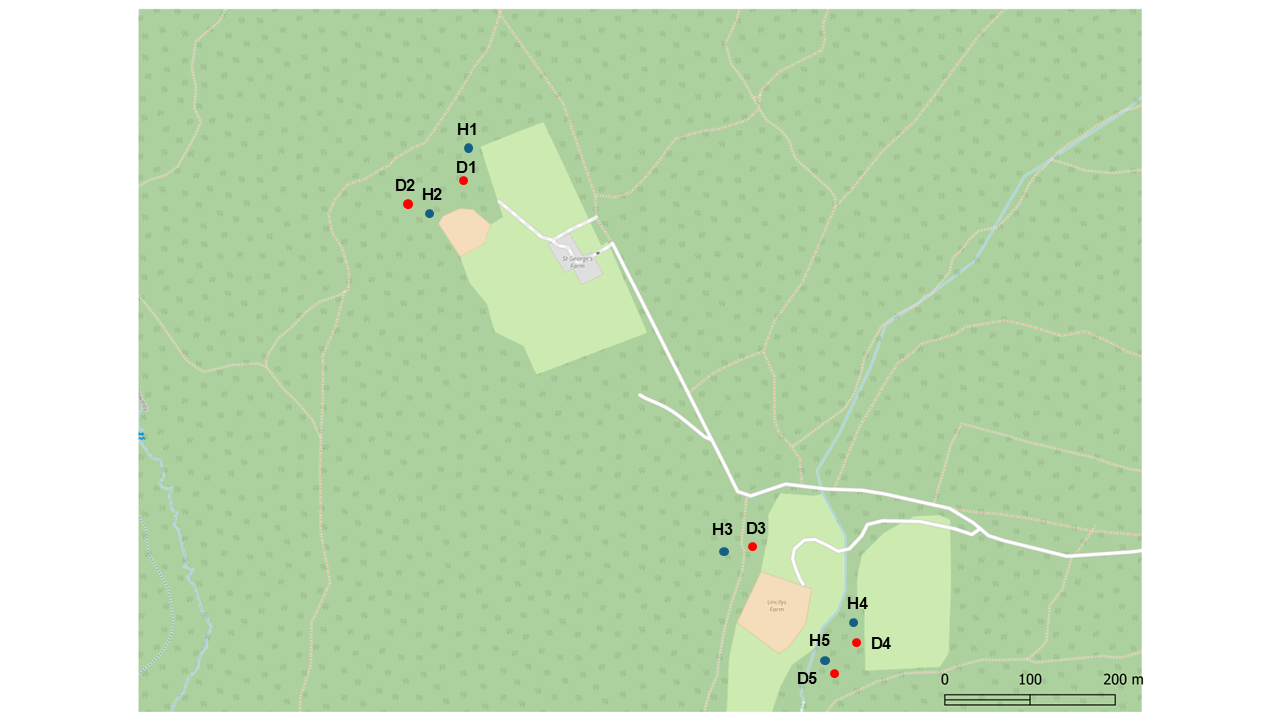


Wyre Forest


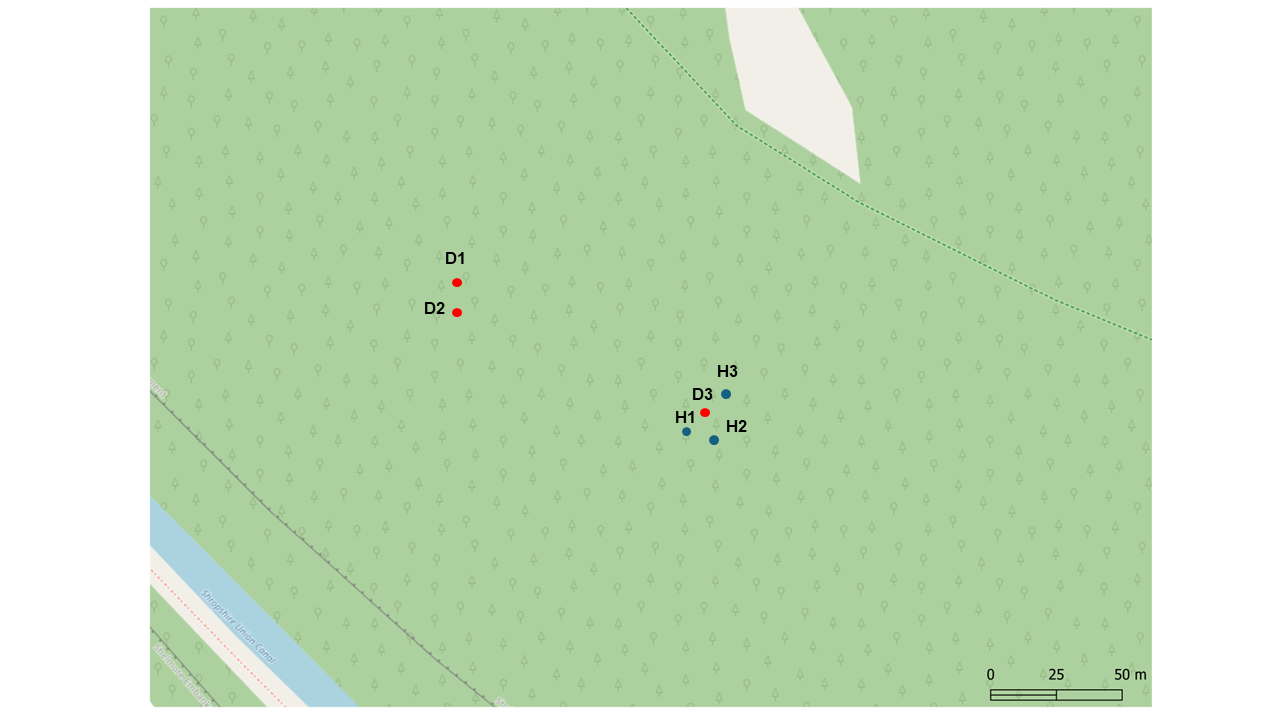


Norbury Park

5.4

(b)

(a)


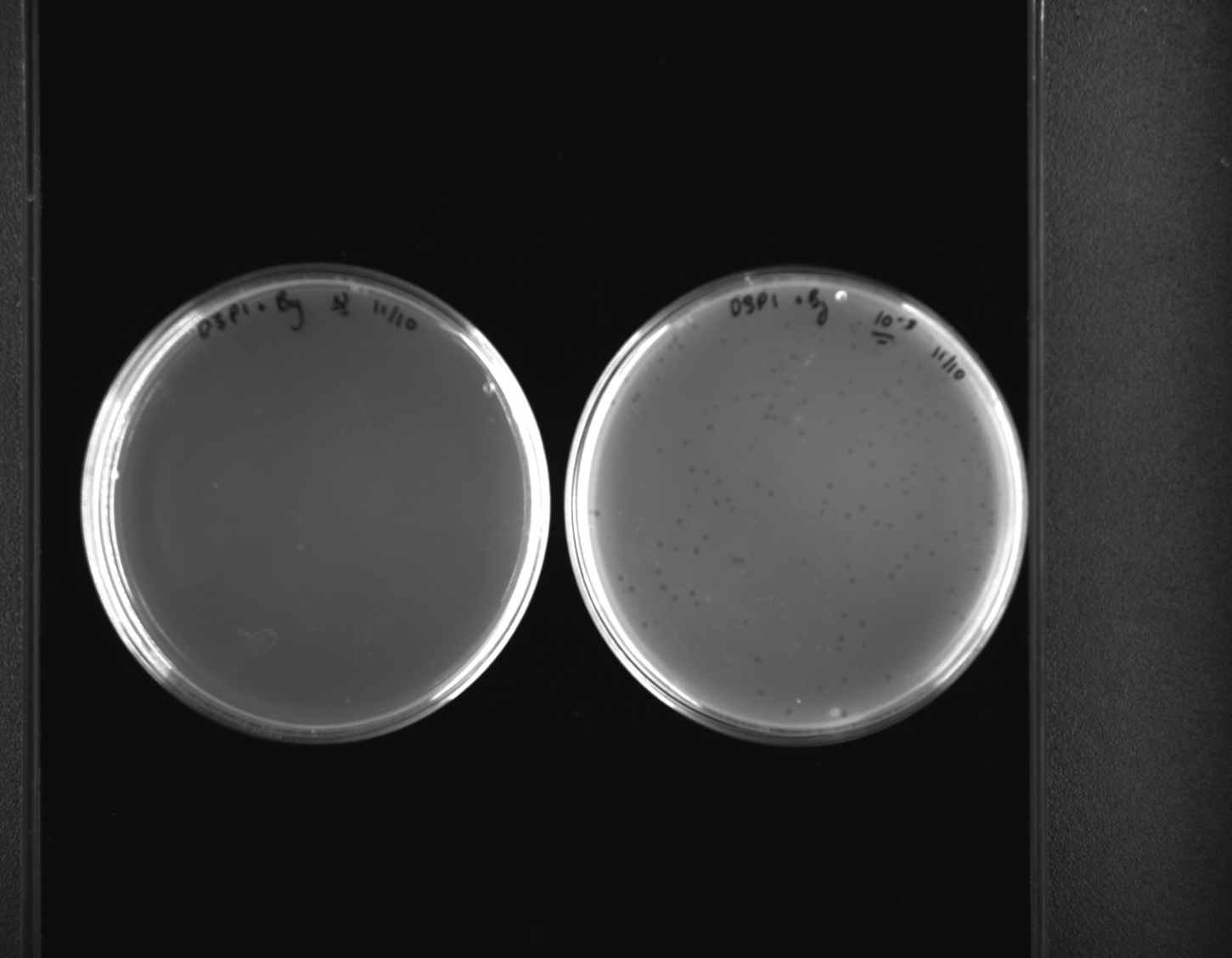

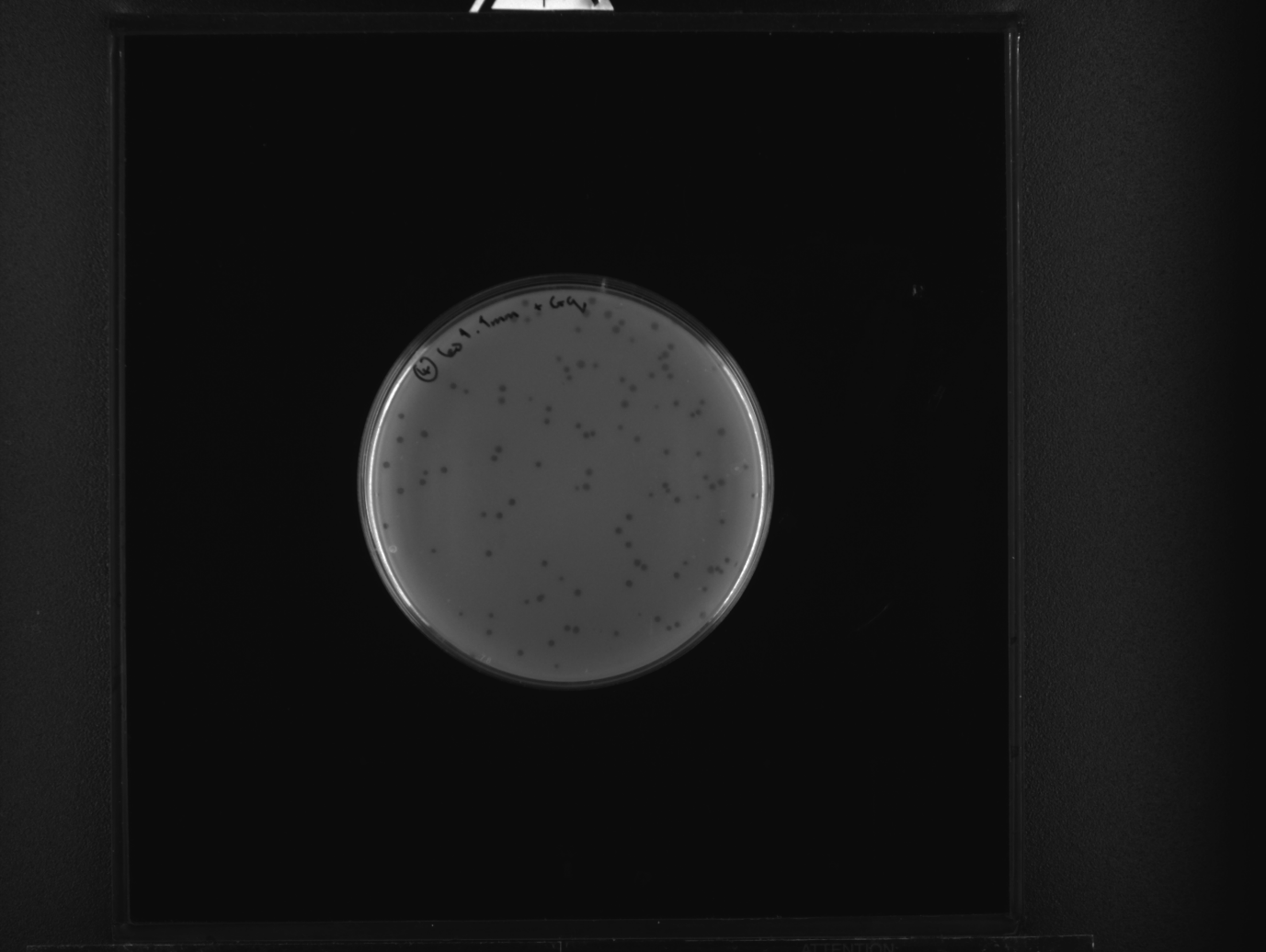


**Supplementary Figure 2.** Plaque morphologies of phage BREN6 on a lawn of *Bg* FRB 141^T^ (a) and of phage GIB1 on a lawn of *Gq* FRB 124 (b).

**Supplementary Table 2.** Host ranges of phages BREN6 and GIB1 are shown. Multiple strains of *Brenneria goodwinii*, *Gibbsiella quercinecans*, other AOD-associated bacteria, and phylogenetically related species were tested for phage susceptibility by spot assay. (+) denotes susceptibility to the phages (plaques arise on bacterial lawn), (-) represents no lysis by the phages (no plaques arise on bacterial lawn), (nt) denotes no test was performed.

| Bacterial species | Strain | BREN6 | GIB1 |
| --- | --- | --- | --- |
| *Brenneria goodwinii* (*Bg*) | FRB 141 | + | - |
|  | FRB 171 | + | - |
|  | FRB 193 | + | nt |
|  | FRB 186 | + | nt |
|  | HTL 1.1b | - | nt |
|  | SOT 2/28a | - | nt |
|  | BH1/28a | - | nt |
|  | DI 16a | - | nt |
|  | BH4/25a | - | nt |
|  | CP82 | - | nt |
|  | Norbury | - | nt |
|  | W14 | + | nt |
|  | W19 | + | nt |
|  | J4.2 | + | nt |
|  | J4.3 | + | nt |
| *Gibbsiella quercinecans* (*Gq*) | FRB 124 | - | + |
|  | FRB 97^T^ | - | + |
|  | AT 186 | nt | + |
|  | FOD 9/25 | nt | + |
|  | HTL 3.3a | nt | + |
|  | HTL 7.16 | nt | - |
|  | Kew 122 | nt | - |
|  | Kew 224 | nt | - |
|  | BHI 656 | nt | - |
|  | BHI 86 | nt | - |
|  | Norbury | nt | - |
|  | W3 | nt | - |
|  | W15 | nt | - |
| *Rahnella victoriana* (*Rv*) | BRK 225^T^ | - | - |
|  | FRB 189 | - | - |
| *Lonsdalea britannica* | 447^T^ | - | - |
| *Raoultella planticola* |  | - | - |
| *Pseudomonas syringae* pv. *syringae* | 9097 | - | - |
| *Brenneria salicis* | 1027^T^ | - | nt |
| *Brenneria roseae* sp. *roseae* | FRB 222^T^ | - | nt |
| *Klebsiella pneumoniae* | LM367 | nt | - |

**Supplementary Table 3.** Phage defence systems identified within the genomes of *Bg* and *Gq* strains.

| Bacteria | Susceptibility to BREN6/GIB1 | Defence system | Proteins | Position (bp) | Strand |
| --- | --- | --- | --- | --- | --- |
| *Bg* FRB 141^T^ | Susceptible | PifA | PifA | 272284 - 274410 | + |
|  |  | PDC-M01 | PDC-M01B | 288972 - 289823 | - |
|  |  |  | PDC-M01A | 289817 - 291568 | - |
|  |  | PARIS | AriA | 3026111 - 3027058 | - |
|  |  |  | AriB | 3027055 - 3028479 | - |
|  |  | DNA modification system | MTase I | 3602404 - 3603348 | - |
|  |  |  | Specificity II | 3603338 - 3604816 | - |
|  |  | AbiE | AbiEi | 3612175 - 3612957 | + |
|  |  |  | AbiEii | 3612944 - 3613873 | + |
|  |  | PD-Lambda-5 | PD-Lambda-5 A | 3761970 - 3763391 | + |
|  |  |  | PD-Lambda-5 B | 3763457 - 3764326 | + |
|  |  | PDC-S49 | PDC-S49 | 4458124 - 4460019 | + |
|  |  | PDC-S07 | PDC-S07 | 5235860 - 5236255 | + |
| *Bg* FRB 171 | Susceptible | AbiE | AbiEii | 43682 - 44611 | - |
|  |  |  | AbiEi | 44598 - 45380 | - |
|  |  | Brex Type I | BrxL | 48192 - 50276 | - |
|  |  |  | PglZ | 50287 - 52884 | - |
|  |  |  | PglX | 53067 - 56711 | - |
|  |  |  | BrxC | 56757 - 60398 | - |
|  |  |  | BrxB | 60410 - 61012 | - |
|  |  |  | BrxA | 61009 - 61617 | - |
|  |  | PsyrTA | PsyrT | 42352 - 44451 | + |
|  |  |  | PsyrA | 44448 - 45764 | + |
|  |  | Mokosh Type II | MkoC | 13033 - 16551 | + |
|  |  | PDC-S39 | PDC-S39 | 60523 - 62403 | + |
|  |  | PDC-M01 | PDC-M01A | 31192 - 32943 | + |
|  |  |  | PDC-M01B | 32937 - 33788 | + |
|  |  | PDC-S07 | PDC-S07 | 61344 - 61739 | + |
|  |  | SoFic | SoFic | 39334 - 40476 | + |
|  |  | CBASS Type III | capP | 551 - 1414 | - |
|  |  |  | capH | 1417 - 1740 | - |
|  |  |  | Cyclase | 1926 - 2891 | + |
|  |  |  | HORMA | 2899 - 3417 | + |
|  |  |  | TRIP13 | 3414 - 4346 | + |
|  |  |  | Effector | 4434 - 5159 | + |
|  |  | Shango | SngC | 5515 - 7713 | - |
|  |  |  | SngB | 7710 - 9026 | - |
|  |  |  | SngA | 9030 - 11339 | - |
| Bg FRB 186 | Susceptible | PDC-M01 | PDC-M01A | 10125 - 11876 | + |
|  |  |  | PDC-M01B | 11870 - 12721 | + |
|  |  | Mokosh_TypeII | MkoC | 139409 - 142927 | - |
|  |  | PsyrTA | PsyrT | 35918 - 38017 | + |
|  |  |  | PsyrA | 38014 - 39330 | + |
|  |  | Brex Type I | BrxA | 68880 - 69488 | + |
|  |  |  | BrxB | 69485 - 70087 | + |
|  |  |  | BrxC | 70099 - 73740 | + |
|  |  |  | PglX | 73786 - 77430 | + |
|  |  |  | PglZ | 77613 - 80210 | + |
|  |  |  | BrxL | 80221 - 82305 | + |
|  |  | AbiE | AbiEi | 85117 - 85899 | + |
|  |  |  | AbiEii | 85886 - 86815 | + |
|  |  | PDC-S07 | PDC-S07 | 191015 - 191410 | + |
|  |  | PDC-S39 | PDC-S39 | 77635 - 79515 | + |
|  |  | CBASS Type III | capP | 429 - 1292 | - |
|  |  |  | capH | 1295 - 1618 | - |
|  |  |  | Cyclase | 1804 - 2769 | + |
|  |  |  | HORMA | 2777 - 3295 | + |
|  |  |  | TRIP13 | 3292 - 4224 | + |
|  |  |  | Effector | 4312 - 5037 | + |
|  |  | Shango | SngC | 5393 - 7591 | - |
|  |  |  | SngB | 7588 - 8904 | - |
|  |  |  | SngA | 8908 - 11217 | - |
|  |  | SoFic | SoFic | 69733 - 70875 | + |
| *Bg* DI 16a | Resistant | PDC-S07 | PDC-S07 | 795976 - 796371 | + |
|  |  | CRISPR Cas type 1 | Cas1f | 887687 - 888031 | + |
|  |  |  | Cas23f | 888045 - 891314 | + |
|  |  |  | Cas8f | 891331 - 892650 | + |
|  |  |  | Cas5f | 892647 - 893594 | + |
|  |  |  | Cas7f | 893612 - 894616 | + |
|  |  |  | Cas6f | 894627 - 895181 | + |
|  |  |  | CRISPR array | 895311 - 898576 | + |
|  |  | PDC-M01 | PDC-M01B | 1155078 - 1155929 | - |
|  |  |  | PDC-M01A | 1155923 - 1157674 | - |
|  |  | Hachiman type I | HamA1 | 120333 - 11204125 | + |
|  |  |  | HamB1 | 1204128 - 1207103 | + |
|  |  | PsyrTA | PsyrA | 1587047 - 1588363 | - |
|  |  |  | PsyrT | 1588360 - 1590459 | - |
|  |  | PDC-M18 | PDC-M18A | 1668926 - 1670011 | + |
|  |  |  | PDC-M18B | 1670018 - 1670533 | + |
|  |  | BstA | BstA | 3099541 - 3100467 | + |
|  |  | PDC-S04 | PDC-S04 | 4242826 - 4243443 | + |
|  |  | Septu type I | PtuB1 00019 | 4243974 - 4244783 | - |
|  |  |  | PtuA100029 | 4244783 - 4246384 | - |
|  |  | DNA modification system | Specificity I 00118 | 4246394 - 4248031 | - |
|  |  |  | MTase I 00009 | 4248031 - 4249566 | - |
|  |  | PDC-S60 | PDC-S60 | 4525382 - 4528006 | - |
|  |  | Restriction modification type II | Control protein 00004 | 4985239 - 4985544 | + |
|  |  |  | Rease II 00073 | 4985537 - 4986355 | + |
|  |  |  | MTase II 00164 | 4986380 - 4987558 | - |
| *Bg* BH 4/25a | Resistant | PDC-S07 | PDC-S07 | 862186 - 862581 | + |
|  |  | CRSIPR-cas type 1-F1 | CRISPR array | 935630 - 936020 | - |
|  |  |  | Cas1f | 936392 - 937372 | + |
|  |  |  | Cas23f | 937369 - 940638 | + |
|  |  |  | Cas8f | 941075 - 942394 | + |
|  |  |  | Cas5f | 942391 - 943338 | + |
|  |  |  | Cas7f | 943356 - 944360 | + |
|  |  |  | Cas6f | 944371 - 944925 | + |
|  |  |  | CRISPR array | 945055 - 951200 | + |
|  |  | PDC-M06 | PDC-M06A | 1426161 - 1426433 | + |
|  |  |  | PDC-M06B | 1426433 - 1426957 | + |
|  |  | DRT class III | RT UG5-nitrilase | 1466958 - 1470089 | + |
|  |  |  | Drt1b | 1470116 - 1470583 | + |
|  |  | Olokun | OloA | 1479070 - 1480788 | + |
|  |  |  | OloB | 1480785 - 1482515 | + |
|  |  | PD-T4-6 | PD-T4-6 | 1879161 - 1880648 | - |
|  |  | Restriction modification Type I | Rease I | 4245689 - 4248742 | + |
|  |  |  | Specificity I | 4248735 - 4249946 | + |
|  |  |  | PrrC | 4249946 - 4251025 | + |
|  |  |  | Mtase I | 4251022 - 4252677 | + |
|  |  | PDC-S11 | PDC-S11 | 4255887 - 4256666 | + |
|  |  | PDC-S29 | PDC-S29 | 4474955 - 4475560 | - |
|  |  | Phosphorothioation system DndABCDE | DndB | 4488371 - 4489456 | + |
|  |  |  | DndC | 4489453 - 4491087 | + |
|  |  |  | DndD | 4491077 - 4493077 | + |
|  |  |  | DndE | 4493077 - 4493430 | + |
|  |  | Phosphorothioation system PT DndFGH | DndH | 4494952 - 4500033 | - |
|  |  |  | DndG | 4500014 - 4501336 | - |
|  |  |  | DndF | 4501341 - 4502990 | - |
|  |  | AbiE | AbiEi | 4590258 - 4591040 | + |
|  |  |  | AbiEii | 4591027 - 4591956 | + |
| Gq FRB 97 | Susceptible | Wadjet Type I | JetD1 | 1642331 - 1643483 | - |
|  |  |  | JetC1 | 1643484 - 1646406 | - |
|  |  |  | JetB1 | 1646768 - 1647425 | - |
|  |  |  | JetA1 | 1647523 - 1648879 | - |
|  |  | Zorya Type I | ZorD1 | 3347170 - 3350299 | - |
|  |  |  | ZorC1 | 3350295 - 3351621 | - |
|  |  |  | ZorB1 | 3351617 - 3352487 | - |
|  |  |  | ZorA1 | 3352483 - 3354520 | - |
|  |  | CBASS Type II | JAB | 3838879 - 3839341 | - |
|  |  |  | E1-E2 | 3839315 - 3841082 | - |
|  |  |  | Cyclase | 3841081 - 3841963 | - |
|  |  |  | Effector | 3842169 - 3842610 | - |
|  |  | AbiU | AbiU | 5072664 - 5074386 | + |
|  |  | CRISPR - Cas Type 1-F1 | Cas23f | 5211844 - 5215123 | + |
|  |  |  | Cas8f | 5215140 - 5216460 | + |
|  |  |  | Cas5f | 5216456 - 5217395 | + |
|  |  |  | Cas7f | 5217412 - 5218423 | + |
|  |  |  | Cas6f | 5218435 - 5218990 | + |
|  |  | PDC - S07 | PDC-S07 | 18911 - 19307 | + |
|  |  |  | PDC-S07 | 1859004 - 1859409 | - |
|  |  | PDC-S26 | PDC-S26 | 2615289 - 2616360 | - |
|  |  | DndFGH | DndF | 3111756 - 3113406 | + |
|  |  |  | DndG | 3113410 - 3114733 | + |
|  |  |  | DndH | 3114713 - 3119798 | + |
|  |  | DndABCDE | DndE | 3124776 - 3125130 | - |
|  |  |  | DndD | 3125129 - 3127139 | - |
|  |  |  | DndC | 3127128 - 3128763 | - |
|  |  |  | DndB | 3128759 - 3129845 | - |
|  |  | Restriction modification Type I | MTase I | 3333874 - 3336235 | + |
|  |  |  | Specificity I | 3336867 - 3337575 | + |
|  |  |  | REase I | 3337571 - 3340820 | + |
|  |  | PDC-S39 | PDC-S39 | 4865690 - 4867502 | + |
|  |  | PD-T4-6 | PD-T4-6 | 4923679 - 4925152 | - |
| Gq FRB 124 | Susceptible | PDC-S07 | PDC-S07 | 656394 - 656799 | + |
|  |  | Wadjet Type I | JetA1 | 848698 - 850195 | + |
|  |  |  | JetB1 | 850200 - 850950 | + |
|  |  |  | JetC1 | 850946 - 854234 | + |
|  |  |  | JetD1 | 854235 - 855387 | + |
|  |  | CBASS Type II | E1-E2 | 923098 - 924274 | - |
|  |  |  | JAB | 924616 - 925045 | - |
|  |  | Shango | SngC | 2103270 - 2105469 | - |
|  |  |  | SngB | 2105465 - 2106782 | - |
|  |  |  | SngA | 2106785 - 2109095 | - |
|  |  | PDC-S07 | PDC-S07 | 2415886 - 2416282 | - |
|  |  | CRISPR - Cas Type 1-F1 | Cas6f | 2759842 - 2760397 | - |
|  |  |  | Cas7f | 2760409 - 2761420 | - |
|  |  |  | Cas5f | 2761437 - 2762376 | - |
|  |  |  | Cas8f | 2762372 - 2763692 | - |
|  |  |  | Cas23f | 2763709 - 2767081 | - |
|  |  | PD-T4-6 | PD-T4-6 | 3055603 - 3057076 | + |
|  |  | Restriction modification Type I | MTase I | 3149174 - 3150491 | - |
|  |  |  | REase I | 3150483 - 3153537 | - |
|  |  |  | MTase I | 3146374 - 3148027 | - |
|  |  |  | REase I | 4688162 - 4691411 | - |
|  |  |  | MTase I | 4691407 - 4692748 | - |
|  |  |  | MTase I | 4692747 - 4695108 | - |
|  |  | PrrC | PrrC | 3148041 - 3149175 | - |
|  |  | PDC-S67 | PDC-S67 | 3158225 - 3158885 | - |
|  |  | Septu Type I | PtuB1 | 3160372 - 3161041 | - |
|  |  |  | PtuA1 | 3161037 - 3162411 | - |
|  |  | DNA-modification system | DrmC | 3163834 - 3164101 | - |
|  |  | Lamassu Family | LmuA | 3164612 - 3165839 | + |
|  |  |  | LmuC | 3165835 - 3166501 | + |
|  |  |  | LmuB | 3166475 - 3168815 | + |
|  |  | PDC-S39 | PDC-S39 | 3173072 - 3174884 | - |
|  |  | PDC-S66 | PDC-S66 | 4162753 - 4163635 | + |
|  |  | Retron type IV | RT VI | 4167512 - 4168613 | - |
|  |  |  | HTH VI | 4168605 - 4168887 | - |
|  |  |  | SP VI | 4168977 - 4169193 | - |
|  |  | DRT | HTH VI | 4539279 - 4539738 | - |
|  |  | Retron type I-B | msr-msd | 4540900 - 4541005 | + |
|  |  |  | RT I-B | 4541162 - 4542113 | + |
|  |  |  | ATPase-Toprim_I-B | 4542109 - 4543474 | + |
|  |  | Zorya type I | ZorA1 | 4674462 - 4676499 | + |
|  |  |  | ZorB1 | 4676495 - 4677365 | + |
|  |  |  | ZorC1 | 4677361 - 4678687 | + |
|  |  |  | ZorD1 | 4678683 - 4681812 | + |
|  |  | PDC-S08 | PDC-S08 | 4900143 - 4901109 | + |
|  |  | PcaI restriction modification | HNH | 4901186 - 4902062 | - |
|  |  | PDC-S26 | PDC-S26 | 5398724 - 5399795 | + |
|  |  | Retron type IX | HEPNIX | 5402781 - 5403342 | + |
|  |  |  | HTH IX | 5403331 - 5404075 | + |
|  |  |  | msr-msd | 5404101 - 5404237 | + |
|  |  |  | RT IX | 5404239 - 5405202 | + |
| Gq HTL 7.16 | Resistant | PDC-S07 | PDC-S07 | 622706 - 623110 | + |
|  |  | CBASS type I | Effector | 682621 - 683553 | - |
|  |  |  | Cyclase | 683557 - 684552 | - |
|  |  | CBASS | E1-E2 | 890625 - 891800 | - |
|  |  |  | JAB | 892143 - 892571 | - |
|  |  | Gabija | GajA | 1123474 - 1125501 | + |
|  |  |  | GajB | 1125498 - 1127174 | + |
|  |  | SoFic | SoFic | 1617969 - 1618760 | + |
|  |  | PDC-S07 | PDC-S07 | 2404391 - 2404786 | - |
|  |  | Mokosh type II | MkoC | 2610869 - 2614210 | + |
|  |  | CRISPR - Cas Type 1-F1 | CRISPR array | 2740667 - 2742915 | - |
|  |  |  | Cas6f | 2743044 - 2743598 | - |
|  |  |  | Cas7f | 2743610 - 2744620 | - |
|  |  |  | Cas5f | 2744638 - 2745576 | - |
|  |  |  | Cas8f | 2745573 - 2746892 | - |
|  |  |  | Cas23f | 2746910 - 2750188 | - |
|  |  | PD-T4-6 | PD-T4-6 | 3025239 - 3026711 | + |
|  |  | Retron type IV | 2TM IV | 3608377 - 3609093 | - |
|  |  |  | RT IV | 3609087 - 3610274 | - |
|  |  |  | msr-msd | 3610300 - 3610397 | - |
|  |  | Septu type I | PtuB1 | 4291331 - 4292119 | - |
|  |  |  | PtuA1 | 4292558 - 4293385 | - |
|  |  | SEFIR | bSEFIR | 4424039 - 4425475 | + |
|  |  | PD-Lambda-2 | PD-Lambda-2_A | 4431589 - 4431849 | + |
|  |  |  | PD-Lambda-2_B | 4431850 - 4433058 | + |
|  |  |  | PD-Lambda-2_C | 4433075 - 4434202 | + |
|  |  | PDC-S02 | PDC-S02 | 4793533 - 4794540 | - |
|  |  | Restriction modification type I | REase I | 4802962 - 4806240 | - |
|  |  | PDC-S05 | PDC-S05 | 4806237 - 4807283 | - |
|  |  | Restriction modification type I | Specificity I | 4807280 - 4808509 | - |
|  |  |  | MTase I | 4808499 - 4810022 | - |
|  |  |  | Specificity I | 4810029 - 4810607 | - |
| Gq Kew 122 | Resistant | PDC-S07 | PDC-S07 | 654327 - 654731 | + |
|  |  | CBASS | E1-E2 | 926885 - 928060 | - |
|  |  |  | JAB | 928403 - 928831 | - |
|  |  | ShosTA | ShosA | 1166309 - 1167247 | + |
|  |  |  | ShosT | 1167250 - 1167891 | + |
|  |  | PDC-S07 | PDC-S07 | 2438732 - 2439127 | - |
|  |  | AVAST type IV | Avs4 | 2656770 - 2661485 | + |
|  |  | Gabija | GajB | 2677116 - 2678273 | - |
|  |  |  | GajA | 2678539 - 2680245 | - |
|  |  | CRISPR - Cas Type 1-F1 | CRISPR array | 2812930 - 2813257 | - |
|  |  |  | Cas6f | 2813387 - 2813941 | - |
|  |  |  | Cas7f | 2813953 - 2814963 | - |
|  |  |  | Cas5f | 2814981 - 2815919 | - |
|  |  |  | Cas8f | 2815916 - 2817235 | - |
|  |  |  | Cas23f | 2817253 - 2820531 | - |
|  |  | PD-T4-6 | PD-T4-6 | 3101723 - 3103195 | + |
|  |  | PDC-S39 | PDC-S39 | 3152499 - 3154310 | - |
|  |  | Restriction modification type HNH | HNH | 4807949 - 4808821 | + |
|  |  | Shedu | SduA | 4811006 - 4812082 | + |
|  |  | 7-deazaguanine modification system | DpdE | 4817791 - 4820898 | + |
|  |  |  | DpdF | 4820898 - 4823378 | + |
|  |  |  | DpdG | 4823365 - 4824240 | + |
|  |  |  | DpdH | 4824237 - 4827434 | + |
|  |  |  | DpdI | 4827431 - 4828279 | + |
|  |  |  | DpdJ | 4828283 - 4832719 | + |
|  |  |  | DpdK | 4832731 - 4833261 | + |
|  |  |  | DpdD | 4833254 - 4835419 | + |
|  |  |  | DpdB | 4835449 - 4836576 | - |
|  |  |  | DpdA | 4836573 **-** 4837844 | - |
|  |  |  | DpdC | 4837897 - 4838748 | - |
|  |  | AVAST type IV | Avs4 | 4940093 - 4945204 | - |
|  |  | PDC-S13 | PDC-S13 | 5343913 - 5344737 | + |

*Bg* strains FRB 141^T^, DI 16a, and BH 4/25a and *Gq* strains FRB 97^T^, FRB 124, HTL 7.16, and Kew 122 were examined, by PADLOC v2.0.0.0 (Payne et al., 2022).

**Supplementary table 4.** Prophages identified within the genomes of *Bg* and *Gq* strains. Prophages were identified using PHASTEST (Wishart et al., 2023).

| Bacterium | No. | Completeness | Region length (Kb) | Total proteins | Region position (bp) | GC (%) |
| --- | --- | --- | --- | --- | --- | --- |
| *Bg* FRB 141^T^ | 1 | Intact | 51.1 | 63 | 1117565 - 1168677 | 49.35 |
|  | 2 | Intact | 33.2 | 48 | 5065985 - 5099204 | 51.88 |
|  | 3 | Intact | 47.2 | 50 | 5313441 - 5360697 | 49.89 |
|  | 4 | Incomplete | 11.7 | 14 | 270 **-** 12054 | 47.73 |
| *Bg* BH 4/25a | 1 | Intact | 22.5 | 34 | 2239404 - 272917 | 56.68 |
|  | 2 | Incomplete | 16.1 | 19 | 1195869 - 121212 | 47.54 |
| *Bg* DI 7.16 | 1 | Intact | 38.7 | 48 | 2093753 - 2132548 | 50.77 |
|  | 2 | Intact | 59.7 | 70 | 3051810 - 3111530 | 52.28 |
|  | 3 | Incomplete | 30.5 | 22 | 4968696 - 4999262 | 49.96 |
| *Gq* FRB 97 | 1 | Incomplete | 10.9 | 12 | 34231 - 45174 | 49.02 |
| *Gq* FRB 124 | 1 | Incomplete | 30.1 | 12 | 2148127 - 2178242 | 52 |
| *Gq* HTL 7.16 | 1 | Intact | 40.7 | 56 | 4442425 - 4483140 | 49.95 |
| *Gq* Kew 122 | 1 | Incomplete | 29.6 | 25 | 1151613 - 1181253 | 49.98 |

**Supplementary figure 3. The phage susceptibility of *Bg* and *Gq* colonies isolated from oak stem saplings 30 days post infection.** *Bg* **(a)** and *Gq* **(b)** colonies were tested against phages BREN6 or GIB1 respectively, or a sterile buffer control, via killing curve assay, and the difference between the final absorbance at 600 nm value of the phage-treated and control-treated killing curves for each colony was calculated. A difference in A600 close to 0.0 is suggestive of phage resistance, and that over 0.1 suggests phages susceptibility. Colonies were isolated from oak saplings 30 days post infection, and were isolated from populations containing either ‘*Bg* + *Gq*’ or ‘*Bg* + *Gq* + BREN6 + GIB1’. Previously generated phage resistant colonies and phage susceptible colonies (*i.e.* wild type *Bg* and *Gq*) are shown as controls. Each point represents an individual colony, with 20 colonies tested per combination and 6 control colonies tested per species.

(a)

(b)

**Supplementary table 5.** The outcome of general linear mixed model (GLMM) and Tukey’s HSD tests used to determine the impact of time, temperature, and replicate number (random effect) on the log transformed number of plaque forming units per millilitre (PFU ml^-1^) formed by BREN6 and GIB1 stocks. Phage stocks were stored in five different temperatures (-20°C, 4°C, 20°C, 27°C, 37°C) and measured after 7 days and 1 year. Post hoc comparisons are shown comparing the original stock PFU ml^-1^ (time 0: T0) to those after 7 days and 1 year.

| **GLMM** | | | |
| --- | --- | --- | --- |
| **BREN6** | | | |
|  | χ² | df | p-value |
| Time | 511.53 | 2 | **<0.0001** |
| Temperature | 0 | 4 | 1 |
| Time x temperature | 3004.04 | 8 | **<0.0001** |
| **GIB1** | | | |
|  | χ² | df | p-value |
| Time | 115.11 | 2 | **<0.0001** |
| Temperature | 0 | 4 | 1 |
| Time x temperature | 625.32 | 8 | **<0.0001** |
| **Tukey’s HSD test** | | | |
| **BREN6** | | | |
|  | Estimate | 95% CI | p-value |
| -20: T0 – 7 days | 3.483 | 3.1733 - 3.79268 | **<0.0001** |
| -20: T0 – 1 year | 2.064 | 1.75432 - 2.37368 | **<0.0001** |
| 4: T0 – 7 days | -0.0191 | -0.32878 - 0.29058 | 0.992 |
| 4: T0 – 1 year | 0.6446 | 0.33492 - 0.95428 | **0.0001** |
| 20: T0 – 7 days | 0.0708 | -0.23888 - 0.38048 | 0.8956 |
| 20: T0 – 1 year | 2.1835 | 1.87382 - 2.49318 | **<0.0001** |
| 27: T0 – 7 days | 0.2561 | -0.05358 - 0.56578 | 0.2381 |
| 27: T0 – 1 year | 4.1941 | 3.88442 - 4.50378 | **<0.0001** |
| 37: T0 – 7 days | 1.1096 | 0.79992 - 1.41928 | **<0.0001** |
| 37: T0 – 1 year | 7.0851 | 6.77542 - 7.39478 | **<0.0001** |
| **GIB1** | | | |
|  | Estimate | 95% CI | p-value |
| -20: T0 – 7 days | 0.167 | -0.576 - 0.909 | 0.8586 |
| -20: T0 – 1 year | 2.667 | 1.891 - 3.442 | **<0.0001** |
| 4: T0 – 7 days | 0.167 | -0.576 - 0.909 | 0.8586 |
| 4: T0 – 1 year | 0.167 | -0.609 - 0.942 | 0.8696 |
| 20: T0 – 7 days | 0.111 | -0.62 - 0.842 | 0.9325 |
| 20: T0 – 1 year | 5.667 | 4.891 - 6.442 | **<0.0001** |
| 27: T0 – 7 days | 1.111 | 0.38 - 1.842 | **0.0011** |
| 27: T0 – 1 year | 5.667 | 4.891 - 6.442 | **<0.0001** |
| 37: T0 – 7 days | 5.667 | 4.935 - 6.398 | **<0.0001** |
| 37: T0 – 1 year | 5.667 | 4.891 - 6.442 | **<0.0001** |

**Supplementary table 6.** The outcome of general linear mixed model (GLMM) and Tukey’s HSD tests used to determine the fixed effects of phage treatment MOI and time, and of replicate number as a random effect, on the absorbance at 600 nm (A600) of *Bg* populations treated with BREN6 and *Gq* populations treated with GIB1. Killing curves were performed over 48 hours, with time extracted every 12 hours. 3 replicate populations were recorded for each MOI.

| **GLMM** | | | |
| --- | --- | --- | --- |
| **BREN6 killing curve** | | | |
|  | χ² | df | p-value |
| Time | 2.534 | 3 | 0.4692 |
| MOI | 78.194 | 3 | **<0.0001** |
| Time x MOI | 101.208 | 9 | **<0.0001** |
| **GIB1 killing curve** | | | |
|  | χ² | df | p-value |
| Time | 5.253 | 3 | 0.1542 |
| MOI | 30.596 | 3 | **<0.0001** |
| Time x MOI | 10.499 | 9 | 0.3117 |
| **Tukey’s HSD test** | | | |
| **BREN6 killing curve** | | | |
|  | Ratio | 95% CI | p-value |
| 12 h: Control – MOI 10^-5^ | 1.66386 | -0.1696 - 0.52 | **<0.0001** |
| 12 h: Control – MOI 10^-6^ | 1.66845 | 0.1211 - 0.81 | **<0.0001** |
| 12 h: Control – MOI 10^-7^ | 1.64262 | 0.3374 - 1.027 | **<0.0001** |
| 12 h: MOI 10^-5^ – MOI 10^-6^ | 0.00459 | -0.0539 - 0.635 | 1 |
| 12 h: MOI 10^-5^ – MOI 10^-7^ | -0.02124 | 0.1624 - 0.852 | 0.9997 |
| 12 h: MOI 10^-6^– MOI 10^-7^ | -0.02583 | -0.1283 - 0.561 | 0.9995 |
| 24 h: Control – MOI 10^-5^ | 1.86708 | 0.1092 - 0.798 | **<0.0001** |
| 24 h: Control – MOI 10^-6^ | 1.87167 | 0.3612 - 1.05 | **<0.0001** |
| 24 h: Control – MOI 10^-7^ | 1.84584 | 0.5596 - 1.249 | **<0.0001** |
| 24 h: MOI 10^-5^ – MOI 10^-6^ | 0.00459 | -0.0925 - 0.597 | 1 |
| 24 h: MOI 10^-5^ – MOI 10^-7^ | -0.02124 | 0.1058 - 0.795 | 0.9997 |
| 24 h: MOI 10^-6^– MOI 10^-7^ | -0.02583 | -0.1462 - 0.543 | 0.9995 |
| 36 h: Control – MOI 10^-5^ | -0.13594 | 0.1136 - 0.803 | 0.9346 |
| 36 h: Control – MOI 10^-6^ | 1.27138 | 0.3894 - 1.079 | **<0.0001** |
| 36 h: Control – MOI 10^-7^ | 0.85072 | 0.4381 - 1.127 | **0.0012** |
| 36 h: MOI 10^-5^ – MOI 10^-6^ | 1.40732 | -0.0687 - 0.62 | **<0.0001** |
| 36 h: MOI 10^-5^ – MOI 10^-7^ | 0.98666 | -0.0201 - 0.669 | **0.0001** |
| 36 h: MOI 10^-6^– MOI 10^-7^ | -0.42066 | -0.2959 - 0.393 | 0.2585 |
| 48 h: Control – MOI 10^-5^ | -0.62879 | 0.0904 - 0.78 | **0.0315** |
| 48 h: Control – MOI 10^-6^ | 0.30401 | 0.4009 - 1.09 | 0.5479 |
| 48 h: Control – MOI 10^-7^ | -0.00368 | 0.2058 - 0.895 | 1 |
| 48 h: MOI 10^-5^ – MOI 10^-6^ | 0.9328 | -0.034 - 0.655 | **0.0003** |
| 48 h: MOI 10^-5^ – MOI 10^-7^ | 0.62511 | -0.2292 - 0.46 | **0.0329** |
| 48 h: MOI 10^-6^– MOI 10^-7^ | -0.30769 | -0.5397 - 0.149 | 0.5376 |
| **GIB1 killing curve** | | | |
|  | Ratio | 95% CI | p-value |
| 12 h: Control – MOI 1 | 0.175 | -0.08764 - 0.43764 | 0.5601 |
| 12 h: Control – MOI 10^-1^ | 0.4657 | 0.20306 - 0.72834 | **0.0029** |
| 12 h: Control – MOI 10^-2^ | 0.682 | 0.41936 - 0.94464 | **<0.0001** |
| 12 h: MOI 1 – MOI 10^-1^ | 0.2907 | 0.02806 - 0.55334 | 0.1324 |
| 12 h: MOI 1 – MOI 10^-2^ | 0.507 | 0.24436 - 0.76964 | **0.0009** |
| 12 h: MOI 10^-1^ – MOI 10^-2^ | 0.2163 | -0.04634 - 0.47894 | 0.3714 |
| 24 h: Control – MOI 1 | 0.4538 | 0.19116 - 0.71644 | **0.004** |
| 24 h: Control – MOI 10^-1^ | 0.7058 | 0.44316 - 0.96844 | **<0.0001** |
| 24 h: Control – MOI 10^-2^ | 0.9041 | 0.64146 - 1.16674 | **<0.0001** |
| 24 h: MOI 1 – MOI 10^-1^ | 0.2521 | -0.01054 - 0.51474 | 0.2369 |
| 24 h: MOI 1 – MOI 10^-2^ | 0.4504 | 0.18776 - 0.71304 | **0.0044** |
| 24 h: MOI 10^-1^ – MOI 10^-2^ | 0.1983 | -0.06434 - 0.46094 | 0.4504 |
| 36 h: Control – MOI 1 | 0.4582 | 0.19556 - 0.72084 | **0.0036** |
| 36 h: Control – MOI 10^-1^ | 0.734 | 0.47136 - 0.99664 | **<0.0001** |
| 36 h: Control – MOI 10^-2^ | 0.7827 | 0.52006 - 1.04534 | **<0.0001** |
| 36 h: MOI 1 – MOI 10^-1^ | 0.2758 | 0.01316 - 0.53844 | 0.1676 |
| 36 h: MOI 1 – MOI 10^-2^ | 0.3245 | 0.06186 - 0.58714 | 0.0734 |
| 36 h: MOI 10^-1^ – MOI 10^-2^ | 0.0487 | -0.21394 - 0.31134 | 0.9837 |
| 48 h: Control – MOI 1 | 0.435 | 0.17236 - 0.69764 | **0.0065** |
| 48 h: Control – MOI 10^-1^ | 0.7455 | 0.48286 - 1.00814 | **<0.0001** |
| 48 h: Control – MOI 10^-2^ | 0.5504 | 0.28776 - 0.81304 | **0.0002** |
| 48 h: MOI 1 – MOI 10^-1^ | 0.3105 | 0.04786 - 0.57314 | 0.0945 |
| 48 h: MOI 1 – MOI 10^-2^ | 0.1154 | -0.14724 - 0.37804 | 0.8252 |
| 48 h: MOI 10^-1^ – MOI 10^-2^ | -0.1951 | -0.45774 - 0.06754 | 0.4651 |

**Supplementary Table 7a.** The outcome of general linear mixed model (GLMM) and Tukey’s HSD tests used to determine the impact of time, bacteria and phage combination, and tree on the log transformed number of colony forming units per millilitre (CFU ml^-1^) formed by *in planta* *Bg* and *Gq* populations. Time and combination were fixed effects, whereas tree number was a random effect. Combinations of either bacteria only (‘Bg+Gq’) or bacteria with phages (‘Bg+Gq+BREN6+GIB1) were applied to the wounds of oak trees, and measurements were taken immediately after inoculation (0 dpi), then at 5, 10 and 30 dpi. Five individual trees (biological replicates) were tested per treatment, with two technical replicates recorded per tree.

| **GLMM** | | | |
| --- | --- | --- | --- |
| ***Bg*** | | | |
|  | χ² | df | p-value |
| Time | 87.9004 | 3 | **<0.0001** |
| Combination | 1.1848 | 1 | 0.2764 |
| Time x Combination | 35.6833 | 3 | **<0.0001** |
| ***Gq*** | | | |
| Time | 49.3072 | 3 | **<0.0001** |
| Combination | 1.8062 | 1 | 0.17897 |
| Time x Combination | 11.2966 | 3 | **0.01023** |
| **Tukey’s HSD test** | | | |
| ***Bg*** | | | |
| Comparison | Estimate | 95% Confidence Interval | p-value |
| 0 dpi: Bg + Gq – Bg + Gq + BREN6 + GIB1 | 0.0742 | - 0.0594 - 0.208 | 0.2764 |
| 5 dpi: Bg + Gq – Bg + Gq + BREN6 + GIB1 | 0.0162 | -0.1173 - 0.15 | 0.812 |
| 10 dpi: Bg + Gq – Bg + Gq + BREN6 + GIB1 | -0.023 | -0.1565 - 0.111 | 0.7359 |
| 30 dpi: Bg + Gq – Bg + Gq + BREN6 + GIB1 | -0.3536 | -0.4871 - 0.22 | **<0.0001** |
| Bg + Gq: 0 dpi – 5 dpi | -0.32795 | -0.4711 - -0.1848 | **<0.0001** |
| Bg + Gq: 0 dpi – 10 dpi | -0.16959 | -0.3128 - -0.0264 | **0.0126** |
| Bg + Gq: 0 dpi – 30 dpi | 0.16604 | 0.0228 - 0.3093 | **0.0154** |
| Bg + Gq: 5 dpi – 10 dpi | 0.15835 | 0.0152 - 0.3016 | **0.0233** |
| Bg + Gq: 5 dpi – 30 dpi | 0.49399 | 0.3508 - 0.6372 | **<0.0001** |
| Bg + Gq: 10 dpi – 30 dpi | 0.33564 | 0.1926 - 0.4787 | **<0.0001** |
| Bg + Gq + BREN6 + GIB1: 0 dpi – 5 dpi | -0.38591 | -0.5292 - -0.2426 | **<0.0001** |
| Bg + Gq + BREN6 + GIB1: 0 dpi – 10 dpi | -0.26674 | -0.4099 - -0.1236 | **<0.0001** |
| Bg + Gq + BREN6 + GIB1: 0 dpi – 30 dpi | -0.2617 | -0.4048 - -0.1186 | **<0.0001** |
| Bg + Gq + BREN6 + GIB1: 5 dpi – 10 dpi | 0.11918 | -0.024 - 0.2624 | 0.1411 |
| Bg + Gq + BREN6 + GIB1: 5 dpi – 30 dpi | 0.12422 | -0.0191 - 0.2675 | 0.1161 |
| Bg + Gq + BREN6 + GIB1: 10 dpi – 30 dpi | 0.00504 | -0.1382 - 0.1483 | 0.9997 |
| ***Gq*** | | | |
| 0 dpi: Bg + Gq – Bg + Gq + BREN6 + GIB1 | 0.0778 | -0.0357 - 0.1912 | 0.179 |
| 5 dpi: Bg + Gq – Bg + Gq + BREN6 + GIB1 | 0.0292 | -0.0842 - 0.1426 | 0.6138 |
| 10 dpi: Bg + Gq – Bg + Gq + BREN6 + GIB1 | 0.0594 | -0.054 - 0.1728 | 0.3046 |
| 30 dpi: Bg + Gq – Bg + Gq + BREN6 + GIB1 | -0.1655 | -0.279 - -0.0521 | **0.0042** |
| Bg + Gq: 0 dpi – 5 dpi | -0.3556 | -0.50424 - -0.2069 | **<0.0001** |
| Bg + Gq: 0 dpi – 10 dpi | -0.2447 | -0.39334 - -0.096 | **0.0001** |
| Bg + Gq: 0 dpi – 30 dpi | -0.0523 | -0.20098 - 0.0964 | 0.8029 |
| Bg + Gq: 5 dpi – 10 dpi | 0.1109 | -0.03778 - 0.2596 | 0.2211 |
| Bg + Gq: 5 dpi – 30 dpi | 0.3033 | 0.15458 - 0.4519 | **<0.0001** |
| Bg + Gq: 10 dpi – 30 dpi | 0.1924 | 0.04369 - 0.341 | **0.0049** |
| Bg + Gq + BREN6 + GIB1: 0 dpi – 5 dpi | -0.4041 | -0.55281 - -0.2555 | **<0.0001** |
| Bg + Gq + BREN6 + GIB1: 0 dpi – 10 dpi | -0.263 | -0.4117 - -0.1143 | **<0.0001** |
| Bg + Gq + BREN6 + GIB1: 0 dpi – 30 dpi | -0.2956 | -0.44429 - -0.1469 | **<0.0001** |
| Bg + Gq + BREN6 + GIB1: 5 dpi – 10 dpi | 0.1411 | -0.00757 - 0.2898 | 0.0701 |
| Bg + Gq + BREN6 + GIB1: 5 dpi – 30 dpi | 0.1085 | -0.04016 - 0.2572 | 0.2388 |
| Bg + Gq + BREN6 + GIB1: 10 dpi – 30 dpi | -0.0326 | -0.18127 - 0.1161 | 0.943 |

**Supplementary table 7b.** The outcome of general linear mixed model (GLMM) and Tukey’s HSD tests used to determine the impact of time and bacteria and phage combination on the log transformed number of plaque forming units per millilitre (PFU ml^-1^) formed by *in planta* BREN6 and GIB1 populations. Time and combination were fixed effects, whereas tree number was a random effect. Combinations of either phage only (‘BREN6+GIB1’) or bacteria with phages (‘Bg+Gq+BREN6+Gq) were applied to the wounds of oak trees, and measurements were taken immediately after inoculation (0 dpi), then at 5, 10 and 30 dpi. Five individual trees (biological replicates) were tested per treatment, with two technical replicates recorded per tree.

| **GLMM** | | | |
| --- | --- | --- | --- |
| **BREN6** | | | |
|  | χ² | df | p-value |
| Time | 6.3131 | 3 | 0.09733 |
| Combination | 0.417 | 1 | 0.51843 |
| Time x Combination | 3.904 | 3 | 0.272023 |
| **GIB1** | | | |
| Time | 2.3359 | 3 | 0.5057 |
| Combination | 0.9931 | 1 | 0.319 |
| Time x Combination | 1.9886 | 3 | 0.5748 |
| **Tukey’s HSD test** | | | |
| **BREN6** | | | |
| Comparison | Estimate | 95% Confidence Interval | p-value |
| 0 dpi: BREN6 + GIB1– Bg + Gq + BREN6 + GIB1 | -0.259 | -1.04 - 0.527 | 0.5184 |
| 5 dpi: BREN6 + GIB1– Bg + Gq + BREN6 + GIB1 | -0.701 | -1.62 - 0.221 | 0.1362 |
| 10 dpi: BREN6 + GIB1– Bg + Gq + BREN6 + GIB1 | -1.681 | -2.97 - -0.397 | **0.0103** |
| 30 dpi: BREN6 + GIB1– Bg + Gq + BREN6 + GIB1 | -21.088 | -26224 - 26182 | 0.9987 |
| BREN6 + GIB1: 0 dpi – 5 dpi | 0.56 | -0.58 – 1.7 | 0.5868 |
| BREN6 + GIB1: 0 dpi – 10 dpi | 1.54 | -0.09 – 3.17 | 0.0731 |
| BREN6 + GIB1 0 dpi – 30 dpi | 21.64 | -34324 - 34367 | 1 |
| BREN6 + GIB1: 5 dpi – 10 dpi | 0.981 | -0.76 – 2.72 | 0.4688 |
| BREN6 + GIB1: 5 dpi – 30 dpi | 21.081 | -34325 – 34367 | 1 |
| BREN6 + GIB1: 10 dpi – 30 dpi | 20.1 | -34326 – 34367 | 1 |
| Bg + Gq + BREN6 + GIB1: 0 dpi – 5 dpi | 0.118 | -0.76 – 1 | 0.9862 |
| Bg + Gq + BREN6 + GIB1: 0 dpi – 10 dpi | 0.118 | -0.76 – 1 | 0.9862 |
| Bg + Gq + BREN6 + GIB1: 0 dpi – 30 dpi | 0.811 | -0.28 – 1.9 | 0.2244 |
| Bg + Gq + BREN6 + GIB1: 5 dpi – 10 dpi | 0 | -0.91 – 0.91 | 1 |
| Bg + Gq + BREN6 + GIB1: 5 dpi – 30 dpi | 0.693 | -0.42 – 1.81 | 0.3781 |
| Bg + Gq + BREN6 + GIB1: 10 dpi – 30 dpi | 0.693 | -0.42 – 1.81 | 0.3781 |
| **GIB1** | | | |
| 0 dpi: BREN6 + GIB1– Bg + Gq + BREN6 + GIB1 | -0.288 | -0.853 - -0.278 | 0.3190 |
| 5 dpi: BREN6 + GIB1– Bg + Gq + BREN6 + GIB1 | -0.869 | -0.152 - -0.221 | **0.0085** |
| 10 dpi: BREN6 + GIB1– Bg + Gq + BREN6 + GIB1 | -0.728 | -0.137 - -0.09 | **0.0252** |
| 30 dpi: BREN6 + GIB1– Bg + Gq + BREN6 + GIB1 | -22.332 | -0.4871 - 0.22 | **<0.0001** |
| BREN6 + GIB1: 0 dpi – 5 dpi | 0.4796 | -0.43 - 1.39 | 0.5253 |
| BREN6 + GIB1: 0 dpi – 10 dpi | 0.4055 | -0.48 - 1.29 | 0.6426 |
| BREN6 + GIB1: 0 dpi – 30 dpi | 22.1578 | -36298 -3634 | 1 |
| BREN6 + GIB1: 5 dpi – 10 dpi | -0.0741 | -1.06 - 0.92 | 0.9975 |
| BREN6 + GIB1: 5 dpi – 30 dpi | 21.6782 | -36299 - 36342 | 1 |
| BREN6 + GIB1: 10 dpi – 30 dpi | 21.7523 | -36299 -36342 | 1 |
| Bg + Gq + BREN6 + GIB1: 0 dpi – 5 dpi | -0.1018 | -0.77 - 0.57 | 0.9798 |
| Bg + Gq + BREN6 + GIB1: 0 dpi – 10 dpi | -0.0351 | -0.72 – 0.65 | 0.9992 |
| Bg + Gq + BREN6 + GIB1: 0 dpi – 30 dpi | 0.1133 | -0.59 – 0.82 | 0.9764 |
| Bg + Gq + BREN6 + GIB1: 5 dpi – 10 dpi | 0.0667 | -0.6 – 0.73 | 0.994 |
| Bg + Gq + BREN6 + GIB1: 5 dpi – 30 dpi | 0.2151 | -0.48 – 0.91 | 0.8543 |
| Bg + Gq + BREN6 + GIB1: 10 dpi – 30 dpi | 0.1484 | -0.55 – 0.85 | 0.9482 |

**Supplementary table 8a.** The outcome of aligned rank transformation analysis of variation (ART ANOVA) and Tukey’s HSD tests used to determine the impact of time and bacteria and phage combination on the log transformed number of colony forming units per millilitre (CFU ml^-1^) formed by *in planta* *Bg* and *Gq* populations. Combinations of either bacteria only (‘Bg+Gq’) or bacteria with phages (‘Bg+Gq+BREN6+Gq) were applied to the wounds of oak trees, and measurements were taken immediately after inoculation (0 dpi), then at 14 dpi. Five individual trees (biological replicates) were tested per treatment, with two technical replicates recorded per tree.

| **ART ANOVA** | | | | |
| --- | --- | --- | --- | --- |
| ***Bg*** | | | | |
|  |  | F value | df | p-value |
| dpi | | 5.138 | 1 | **0.03** |
| Combination | | 4.262 | 5 | **0.0003** |
| dpi : combination | | 3.251 | 5 | **0.013** |
| ***Gq*** | | | | |
| dpi | | 133.648 | 1 | **<0.0001** |
| Combination | | 10.162 | 5 | **<0.0001** |
| dpi : combination | | 11.544 | 5 | **<0.0001** |
| **Tukey’s HSD test** | | | | |
| ***Bg*** | | | | |
|  | Comparison | Rank difference | 95% Confidence Interval | p-value |
| 0 dpi: Bg - Bg+BREN6 | | -7.1 | -24.5832 - 10.3832 | 0.9996 |
| 0 dpi: Bg - Bg+Gq | | 1.8 | -15.6832 - 19.2832 | 1 |
| 0 dpi: Bg - Bg+Gq+BREN6 | | 4.4 | -13.0832 - 21.8832 | 1 |
| 0 dpi: Bg - Bg+Gq+BREN6+GIB1 | | 10.2 | -7.2832 - 27.6832 | 0.9907 |
| 0 dpi: Bg - Bg+Gq+GIB1 | | 8.6 | -8.8832 - 26.0832 | 0.9978 |
| 0 dpi: Bg+BREN6 - Bg+Gq | | 8.9 | -8.5832 - 26.3832 | 0.997 |
| 0 dpi: Bg+BREN6 - Bg+Gq+BREN6 | | 11.5 | -5.9832 - 28.9832 | 0.9766 |
| 0 dpi: Bg+BREN6 - Bg+Gq+BREN6+GIB1 | | 17.3 | -0.1832 - 34.7832 | 0.7289 |
| 0 dpi: Bg+BREN6 - Bg+Gq+GIB1 | | 15.7 | -1.7832 - 33.1832 | 0.8303 |
| 0 dpi: Bg+Gq - Bg+Gq+BREN6 | | 2.6 | -14.8832 - 20.0832 | 1 |
| 0 dpi: Bg+Gq - Bg+Gq+BREN6+GIB1 | | 8.4 | -9.0832 - 25.8832 | 0.9982 |
| 0 dpi: Bg+Gq - Bg+Gq+GIB1 | | 6.8 | -10.6832 - 24.2832 | 0.9997 |
| 0 dpi: Bg+Gq+BREN6 - Bg+Gq+BREN6+GIB1 | | 5.8 | -11.6832 - 23.2832 | 0.9999 |
| 0 dpi: Bg+Gq+BREN6 - Bg+Gq+GIB1 | | 4.2 | -13.2832 - 21.6832 | 1 |
| 0 dpi: Bg+Gq+BREN6+GIB1 - Bg+Gq+GIB1 | | -1.6 | -19.0832 - 15.8832 | 1 |
| 14 dpi: Bg - Bg+BREN6 | | 12.3 | -5.1832 - 29.7832 | 0.962 |
| 14 dpi: Bg - Bg+Gq | | 42.4 | 24.9168 - 59.8832 | **0.001** |
| 14 dpi: Bg - Bg+Gq+BREN6 | | 35 | 17.5168 - 52.4832 | **0.0131** |
| 14 dpi: Bg - Bg+Gq+BREN6+GIB1 | | 34.8 | 17.3168 - 52.2832 | **0.0139** |
| 14 dpi: Bg - Bg+Gq+GIB1 | | 34 | 16.5168 - 51.4832 | **0.018** |
| 14 dpi: Bg+BREN6 - Bg+Gq | | 30.1 | 12.6168 - 47.5832 | 0.0579 |
| 14 dpi: Bg+BREN6 - Bg+Gq+BREN6 | | 22.7 | 5.2168 - 40.1832 | 0.3381 |
| 14 dpi: Bg+BREN6 - Bg+Gq+BREN6+GIB1 | | 22.5 | 5.0168 - 39.9832 | 0.3509 |
| 14 dpi: Bg+BREN6 - Bg+Gq+GIB1 | | 21.7 | 4.2168 - 39.1832 | 0.4046 |
| 14 dpi: Bg+Gq - Bg+Gq+BREN6 | | -7.4 | -24.8832 - 10.0832 | 0.9994 |
| 14 dpi: Bg+Gq - Bg+Gq+BREN6+GIB1 | | -7.6 | -25.0832 - 9.8832 | 0.9993 |
| 14 dpi: Bg+Gq - Bg+Gq+GIB1 | | -8.4 | -25.8832 - 9.0832 | 0.9982 |
| 14 dpi: Bg+Gq+BREN6 - Bg+Gq+BREN6+GIB1 | | -0.2 | -17.6832 - 17.2832 | 1 |
| 14 dpi: Bg+Gq+BREN6 - Bg+Gq+GIB1 | | -1 | -18.4832 - 16.4832 | 1 |
| 14 dpi: Bg+Gq+BREN6+GIB1 - Bg+Gq+GIB1 | | -0.8 | -18.2832 - 16.6832 | 1 |
| Bg: 0 dpi - 14 dpi | | -17.2 | -34.6832 - 0.2832 | 0.7358 |
| Bg+BREN6: 0 dpi v 14 dpi | | 2.2 | -15.2832 - 19.6832 | 1 |
| Bg+Gq: 0 dpi v 14 dpi | | 23.4 | 5.9168 - 40.8832 | 0.2954 |
| Bg+Gq+BREN6: 0 dpi v 14 dpi | | 13.4 | -4.0832 - 30.8832 | 0.9326 |
| Bg+Gq+GIB1: 0 dpi v 14 dpi | | 8.2 | -9.2832 - 25.6832 | 0.9986 |
| Bg+Gq+BREN6 +GIB1: 0 dpi v 14 dpi | | 7.4 | -10.0832 - 24.8832 | 0.9994 |
| ***Gq*** | | | | |
| 0 dpi: Bg+Gq -Bg+Gq+BREN6 | | 7.4 | -1.7336 - 16.5336 | 0.9054 |
| 0 dpi: Bg+Gq - Bg+Gq+BREN6+GIB1 | | 21.6 | 12.4664 - 30.7336 | **0.0015** |
| 0 dpi: Bg+Gq - Bg+Gq+GIB1 | | 12.3 | 3.1664 - 21.4336 | 0.2884 |
| 0 dpi: Bg+Gq – Gq | | 4.6 | -4.5336 - 13.7336 | 0.9973 |
| 0 dpi: Bg+Gq - Gq+GIB1 | | 15 | 5.8664 - 24.1336 | 0.0855 |
| 0 dpi: Bg+Gq+BREN6 - Bg+Gq+BREN6+GIB1 | | 10.56 | 1.4264 - 19.6936 | 0.127 |
| 0 dpi: Bg+Gq+BREN6 - Bg+Gq+GIB1 | | 10.38 | 1.2464 - 19.5136 | 0.9954 |
| 0 dpi: Bg+Gq+BREN6 - Gq | | 10.2 | 1.0664 - 19.3336 | 1 |
| 0 dpi: Bg+Gq+BREN6 - Gq+GIB1 | | 10.02 | 0.8864 - 19.1536 | 0.8892 |
| 0 dpi: Bg+Gq+GIB1 - Gq | | 7.14 | -1.9936 - 16.2736 | 0.8806 |
| 0 dpi: Bg+Gq+GIB1 - Gq+GIB1 | | 6.96 | -2.1736 - 16.0936 | 1 |
| 0 dpi: Gq - Gq+GIB1 | | 5.7 | -3.4336 - 14.8336 | 0.5372 |
| 14 dpi: Bg+Gq - Bg+Gq+BREN6 | | 3.36 | -5.7736 - 12.4936 | 0.3719 |
| 14 dpi: Bg+Gq - Bg+Gq+BREN6+GIB1 | | 3.18 | -5.9536 - 12.3136 | 1 |
| 14 dpi: Bg+Gq - Bg+Gq+GIB1 | | 3 | -6.1336 - 12.1336 | 1 |
| 14 dpi: Bg+Gq - Gq | | 2.82 | -6.3136 - 11.9536 | 1 |
| 14 dpi: Bg+Gq - Gq+GIB1 | | 2.64 | -6.4936 - 11.7736 | 0.9994 |
| 14 dpi: Bg+Gq+BREN6- Bg+Gq+BREN6+GIB1 | | 2.46 | -6.6736 - 11.5936 | 0.3847 |
| 14 dpi: Bg+Gq+BREN6 - Bg+Gq+GIB1 | | 2.28 | -6.8536 - 11.4136 | 0.2669 |
| 14 dpi: Bg+Gq+BREN6 - Gq | | 2.1 | -7.0336 - 11.2336 | 0.1833 |
| 14 dpi: Bg+Gq+BREN6 - Gq+GIB1 | | 1.92 | -7.2136 - 11.0536 | 0.8806 |
| 14 dpi: Bg+Gq+BREN6+GIB1 - Bg+Gq+GIB1 | | 1.74 | -7.3936 - 10.8736 | 1 |
| 14 dpi: Bg+Gq+BREN6+GIB1 - Gq | | 1.56 | -7.5736 - 10.6936 | 1 |
| 14 dpi: Bg+Gq+BREN6+GIB1 - Gq+GIB1 | | 1.38 | -7.7536 - 10.5136 | 0.9995 |
| 14 dpi: Bg+Gq+GIB1 - Gq | | 1.2 | -7.9336 - 10.3336 | 1 |
| 14 dpi: Bg+Gq+GIB1 - Gq+GIB1 | | 1.02 | -8.1136 - 10.1536 | 0.9961 |
| 14 dpi: Gq- Gq+GIB1 | | 0.84 | -8.2936 - 9.9736 | 0.9844 |
| Gq: 0 dpi – 14 dpi | | 4.8 | -4.3336 - 13.9336 | **0.0039** |
| Gq+GIB1: 0 dpi – 14 dpi | | 3.54 | -5.5936 - 12.6736 | **<0.0001** |
| Bg+Gq: 0 dpi – 14 dpi | | 9.66 | 0.5264 - 18.7936 | **<0.0001** |
| Bg+Gq+BREN6: 0 dpi – 14 dpi | | 6.24 | -2.8936 - 15.3736 | **<0.0001** |
| Bg+Gq+GIB1: 0 dpi – 14 dpi | | 7.86 | -1.2736 - 16.9936 | **<0.0001** |

**Supplementary table 8b.** The outcome of aligned rank transformation analysis of variation (ART ANOVA) and Tukey’s HSD tests used to determine the impact of time and bacteria and phage combination on the log transformed number of plaque forming units per millilitre (CFU ml^-1^) formed by *in planta* BREN6 and GIB1 populations. Combinations of either bacteria only (‘Bg+Gq’) or bacteria with phages (‘Bg+Gq+BREN6+Gq) were applied to the wounds of oak trees, and measurements were taken immediately after inoculation (0 dpi), then at 14 dpi. Five individual trees (biological replicates) were tested per treatment, with two technical replicates recorded per tree.

| **ART ANOVA** | | | | |
| --- | --- | --- | --- | --- |
| **BREN6** | | | | |
|  |  | F value | df | p-value |
| dpi | | 130.2518 | 1 | **<0.0001** |
| Combination | | 8.0647 | 4 | **<0.0001** |
| dpi : combination | | 1.1508 | 4 | 0.3469 |
| **GIB1** | | | | |
| dpi | | 126.58 | 1 | **<0.0001** |
| Combination | | 30.69 | 4 | **<0.0001** |
| dpi : combination | | 18.247 | 4 | **<0.0001** |
| **Tukey’s HSD test** | | | | |
| Comparison | | Rank difference | 95% Confidence Interval | p-value |
| 0 dpi: Bg+BREN6 - Bg+Gq+BREN6 | | 8 | 0.846 - 15.154 | 0.4785 |
| 0 dpi: Bg+BREN6 v Bg+Gq+BREN6+GIB1 | | 2.6 | -4.554 - 9.754 | 0.9993 |
| 0 dpi: Bg+BREN6 v BREN6 | | 20.2 | 13.046 - 27.354 | **0.0001** |
| 0 dpi: Bg+BREN6 v BREN6+GIB1 | | 15 | 7.846 - 22.154 | **0.0065** |
| 0 dpi: Bg+Gq+BREN6 v Bg+Gq+BREN6+GIB1 | | -5.4 | -12.554 - 1.754 | 0.8923 |
| 0 dpi: Bg+Gq+BREN6 v BREN6 | | 12.2 | 5.046 - 19.354 | 0.0506 |
| 0 dpi: Bg+Gq+BREN6 v BREN6+GIB1 | | 7 | -0.154 - 14.154 | 0.6575 |
| 0 dpi: Bg+Gq+BREN6+GIB1 v BREN6 | | 17.6 | 10.446 - 24.754 | **0.0008** |
| 0 dpi: Bg+Gq+BREN6+GIB1 v BREN6+GIB1 | | 12.4 | 5.246 - 19.554 | **0.0442** |
| 0 dpi: BREN6 - BREN6+GIB1 | | -5.2 | -12.354 - 1.954 | 1 |
| 14 dpi: Bg+BREN6 - Bg+Gq+BREN6 | | -1.7 | -8.854 - 5.454 | 0.9955 |
| 14 dpi: Bg+BREN6 - Bg+Gq+BREN6+GIB1 | | -3.3 | -10.454 - 3.854 | 0.4611 |
| 14 dpi: Bg+BREN6 - BREN6 | | 8.1 | 0.946 - 15.254 | 0.4611 |
| 14 dpi: Bg+BREN6 - BREN6+GIB1 | | 8.1 | 0.946 - 15.254 | 1 |
| 14 dpi: Bg+Gq+BREN6 - Bg+Gq+BREN6+GIB1 | | -1.6 | -8.754 - 5.554 | 0.2143 |
| 14 dpi: Bg+Gq+BREN6 - BREN6 | | 9.8 | 2.646 - 16.954 | 0.2143 |
| 14 dpi: Bg+Gq+BREN6 - BREN6+GIB1 | | 9.8 | 2.646 - 16.954 | 0.0852 |
| 14 dpi: Bg+Gq+BREN6+GIB1 - BREN6 | | 11.4 | 4.246 - 18.554 | 0.0852 |
| 14 dpi: Bg+Gq+BREN6+GIB1 - BREN6+GIB1 | | 11.4 | 4.246 - 18.554 | 1 |
| 14 dpi: BREN6 - BREN6+GIB1 | | -1.7 | -8.854 - 5.454 | 0.9955 |
| BREN6: 0 dpi – 14 dpi | | 18.1 | 10.946 - 25.254 | **0.0005** |
| BREN6+GIB1: 0 dpi – 14 dpi | | 23.3 | 16.146 - 30.454 | **<0.0001** |
| Bg+BREN6: 0 dpi - 14 dpi | | 30.2 | 23.046 - 37.354 | **<0.0001** |
| Bg+Gq+BREN6: 0 dpi – 14 dpi | | 20.5 | 13.346 - 27.654 | **0.0001** |
| Bg+Gq+BREN6+GIB1: 0 dpi – 14 dpi | | 24.3 | 17.146 - 31.454 | **<0.0001** |
| **GIB1** | | | | |
| 0 dpi: Gq+GIB1 - Bg+Gq+BREN6+GIB1 | | -27.2 | -34.0404 - -20.3596 | **<0.0001** |
| 0 dpi: Gq+GIB1 - Bg+Gq+GIB1 | | -23.5 | -30.3404 - -16.6596 | **<0.0001** |
| 0 dpi: Gq+GIB1 - GIB1 | | -3.8 | -10.6404 - 3.0404 | 0.983 |
| 0 dpi: Gq+GIB1 - Gq+GIB1 | | -26.7 | -33.5404 - -19.8596 | **<0.0001** |
| 0 dpi: Bg+Gq+BREN6+GIB1 - Bg+Gq+GIB1 | | 3.7 | -3.1404 - 10.5404 | 0.9858 |
| 0 dpi: Bg+Gq+BREN6+GIB1 - GIB1 | | 23.4 | 16.5596 - 30.2404 | **<0.0001** |
| 0 dpi: Bg+Gq+BREN6+GIB1 - Gq+GIB1 | | 0.5 | -6.3404 - 7.3404 | 1 |
| 0 dpi: GIB1 - Gq+GIB1 | | -22.9 | -29.7404 - -16.0596 | **<0.0001** |
| 14 dpi: Gq+GIB1 - Bg+Gq+BREN6+GIB1 | | -18.8 | -25.6404 - -11.9596 | **0.0001** |
| 14 dpi: Gq+GIB1 - Bg+Gq+GIB1 | | -19.3 | -26.1404 - -12.4596 | **0.0001** |
| 14 dpi: Gq+GIB1 - GIB1 | | 0 | -6.8404 - 6.8404 | 1 |
| 14 dpi: Gq+GIB1 - Gq+GIB1 | | -22.2 | -29.0404 - -15.3596 | **<0.0001** |
| 14 dpi: Bg+Gq+BREN6+GIB1 - Bg+Gq+GIB1 | | -0.5 | -7.3404 - 6.3404 | 1 |
| 14 dpi: Bg+Gq+BREN6+GIB1 - GIB1 | | 18.8 | 11.9596 - 25.6404 | **0.0001** |
| 14 dpi: Bg+Gq+BREN6+GIB1 - Gq+GIB1 | | -3.4 | -10.2404 - 3.4404 | 0.9921 |
| 14 dpi: Bg+Gq+GIB1 - GIB1 | | 19.3 | 12.4596 - 26.1404 | **0.0001** |
| 14 dpi: Bg+Gq+GIB1 - Gq+GIB1 | | -2.9 | -9.7404 - 3.9404 | 0.9976 |
| 14 dpi: GIB1 - Gq+GIB1 | | -22.2 | -29.0404 - -15.3596 | **<0.0001** |
| GIB1: 0 dpi – 14 dpi | | 11.7 | 4.8596 - 18.5404 | **0.049** |
| BREN6+GIB1: 0 dpi – 14 dpi | | 20.1 | 13.2596 - 26.9404 | **<0.0001** |
| Gq+GIB1: 0 dpi - 14 dpi | | 15.9 | 9.0596 - 22.7404 | **0.0017** |
| Bg+Gq+GIB1: 0 dpi – 14 dpi | | 15.5 | 8.6596 - 22.3404 | **0.0025** |
| Bg+Gq+BREN6+GIB1: 0 dpi – 14 dpi | | 16.2 | 9.3596 - 23.0404 | **0.0013** |
